# INHAT subunit SET/TAF-Iβ regulates PRC1-independent H2AK119 mono-ubiquitination via E3 ligase MIB1 in colon cancer

**DOI:** 10.1093/narcan/zcad050

**Published:** 2023-09-22

**Authors:** Junyoung Park, Ji-Young Kim, Jin Woo Park, Joo Young Kang, Hyein Oh, Ja Young Hahm, Yun-Cheol Chae, Debabrata Chakravarti, Sang Beom Seo

**Affiliations:** Department of Life Science, College of Natural Sciences, Chung-Ang University, Seoul 06974, Republic of Korea; Department of Life Science, College of Natural Sciences, Chung-Ang University, Seoul 06974, Republic of Korea; Department of Life Science, College of Natural Sciences, Chung-Ang University, Seoul 06974, Republic of Korea; Department of Life Science, College of Natural Sciences, Chung-Ang University, Seoul 06974, Republic of Korea; Department of Life Science, College of Natural Sciences, Chung-Ang University, Seoul 06974, Republic of Korea; Department of Life Science, College of Natural Sciences, Chung-Ang University, Seoul 06974, Republic of Korea; Department of Life Science, College of Natural Sciences, Chung-Ang University, Seoul 06974, Republic of Korea; Division of Reproductive Sciences in Medicine, Department of Obstetrics and Gynecology, Northwestern University Feinberg School of Medicine, Chicago, IL 60611, USA; Department of Life Science, College of Natural Sciences, Chung-Ang University, Seoul 06974, Republic of Korea

## Abstract

SET/TAF-Iβ, a subunit of the inhibitor of acetyltransferases (INHAT) complex, exhibits transcriptional repression activity by inhibiting histone acetylation. We find that SET/TAF-Iβ regulates mono-ubiquitination of histone H2A at lysine 119 (H2AK119ub), which is involved in polycomb-mediated transcriptional repression, in HCT116 cells. In this report, we demonstrate that SET/TAF-Iβ acts as an E2 ubiquitin-conjugating enzyme for PRC1-independent H2AK119ub. Furthermore, we identify that MIB1 is the E3 ligase partner for SET/TAF-Iβ using LC-MS/MS and *in vitro* ubiquitination assays. Transcriptome analysis reveals that SET/TAF-Iβ and MIB1 regulate the expression of genes related to DNA replication and cell cycle progression in HCT116 cells, and knockdown of either protein reduces proliferation of HCT116 cells by impeding cell cycle progression. Together, our study reveals a novel PRC1-independent epigenetic regulatory mechanism for H2AK119ub by SET/TAF-Iβ and MIB1 in colon cancer.

## Graphical Abstract

**Graphical Abstract:**
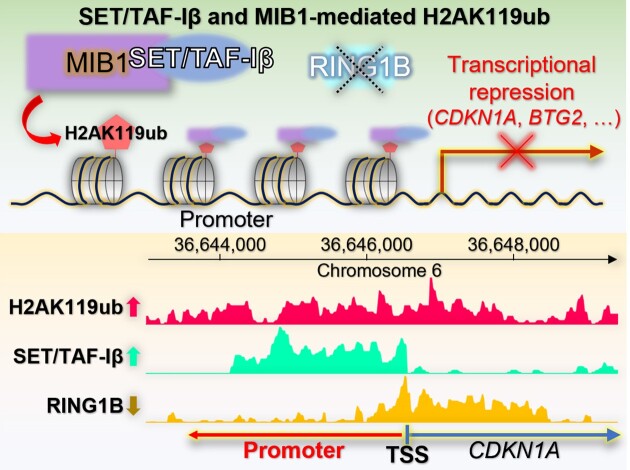

## INTRODUCTION

As a highly acidic domain-containing protein, SET/TAF-Iβ has been previously identified as a subunit of the inhibitor of acetyltransferases (INHAT) complex, which binds to histones and prevents acetylation by p300/CBP and PCAF (1). SET/TAF-Iβ specifically binds to unacetylated and hypo-acetylated histones, which implies an *in vivo* role in transcriptional repression (2). Multitasking protein SET/TAF-Iβ blocks both p53-mediated cell cycle arrest and apoptosis in response to cellular stress by inhibiting p53 acetylation (3). In fact, SET/TAF-Iβ was originally identified as a translocated gene in acute undifferentiated leukemia, which further supports its oncogenic activity (4–6). Recent studies suggest that acidic domain-containing proteins, including SET/TAF-Iβ, can regulate the activities of proteins with cluster of lysine residues such as p53 in an acetylation-modulated manner (7).

The mono-ubiquitinated histone H2A lysine 119 (H2AK119ub) is a highly conserved histone modification found in eukaryotic organisms (8). The mono-ubiquitinated residue was mapped to K119 and initially speculated to be involved in transcriptional regulation (8,9). The polycomb repressive complex 1 (PRC1) has been identified as a major regulator of this modification, suggesting a role for H2AK119ub in gene repression. Specifically, an E3 ubiquitin-protein ligase RING2 (RING1B) along with polycomb complex protein BMI-1, which is a polycomb-group protein (PcG) subunit, make up the core heterodimer of PRC1 that catalyzes the H2AK119ub. Moreover, H2AK119ub colocalizes with H3K27me3, a transcription repressive modification catalyzed by the polycomb repressive complex 2 (PRC2) (10).

We found that SET/TAF-Iβ initially recognizes mono-, di-, and tri-methylated H3 lysine 27 (H3K27me1/2/3) modification formed by PRC2 and is recruited to the target gene promoter to foster another layer of transcriptional repression (11). The fact that SET/TAF-Iβ is required for the establishment of silencing of target genes in various tumor cells raise questions about the contribution of SET/TAF-Iβ in transcriptional repression regulatory mechanisms independent of PRC1-mediated H2AK119ub (1,2).

Mind bomb homolog 1 (MIB1), an E3 ubiquitin ligase, is highly conserved across species and abundantly expressed in both embryonic and adult tissues in vertebrates (12). *Mib1* knockout mice are embryonic lethal and exhibit a neurogenic phenotype as well as pleiotropic Notch-related defects in embryo suggesting the possible role in the regulation of multiple Dll and Jag ligands in development (13). It has been suggested that MIB1 has a dynamic protein interactome network involved in both antiviral innate immune responses and neuronal development (14,15). Previous studies have also reported a connection between Notch signaling pathway and DNA replication process in *Caenorhabditis elegans* suggesting that subunits of DNA polymerase α-primase complex are regulated by GLP-1/Notch signaling pathway (16).

Here, we first discovered a novel role of SET/TAF-Iβ as an E2 ubiquitin-conjugating enzyme and characterized its function as an important regulator of H2AK119ub-mediated transcriptional regulation. We found that MIB1 is an E3 ligase partner for SET/TAF-Iβ and induces H2AK119ub-mediated transcriptional regulation in HCT116 cells. Also, mono-ubiquitination of histone H2AK119 by SET/TAF-Iβ and MIB1 was independent of PRC1. Moreover, we demonstrate that knockdown of SET/TAF-Iβ and MIB1 remarkably inhibits proliferation of HCT116 cells by interrupting DNA replication and eventually delaying cell cycle progression, respectively.

## MATERIALS AND METHODS

### Cell culture and transfection

HCT116 (purchased from Korean Cell Line Bank; KCLB, Seoul, Korea) and NCI-H1299 (KCLB) cells were grown in RPMI-1640 (Gibco, Waltham, MA, USA) supplemented with 10% heat-inactivated fetal bovine serum (Gibco) and 1% penicillin-streptomycin (Welgene, Gyeongsan, Korea) at 37°C in an incubator with 5% CO_2_. MCF-7 (KCLB), HeLa (KCLB) and HEK293T (purchased from American Type Culture Collection; ATCC, Manassas, VA, USA) cells were grown in DMEM (Gibco) under the same conditions without contamination with mycoplasma. For transfection, polyethylenimine (PEI) (Polysciences, Warrington, PA, USA) or jetOPTIMUS^®^ (Polyplus, Illkirch, France) was used. Cells were harvested after 48 h after transfection and used in subsequent experiments.

### Stable knockdown cell lines

Target sequences of the short hairpin RNA (shRNA) for SET/TAF-Iβ, TRIM21, and MIB1 were 5′-GCTGAAATTAAATGCCACTTT-3′, 5′-GAGTTGGCTGAGAAGTTGGAA-3′, and 5′-CCTCTGGGATAATGGTGCTAA-3′, respectively. The oligonucleotides containing target sequences were annealed and inserted into the AgeI/EcoRI site of the pLKO.1-TRC plasmid (#10878, Addgene, Watertown, MA, USA). Co-transfection of the cloned pLKO.1-TRC vector with VSV-G envelope expressing plasmid pMD2.G (#12259, Addgene) and lentiviral packaging plasmid psPAX2 (#12260, Addgene) into HEK293T cells was performed. Supernatants were harvested 2 and 3 days after transfection and used for lentiviral transduction in the presence of 8 μg/ml hexadimethrine bromide (Sigma-Aldrich, St. Louis, MO, USA). Puromycin (Sigma-Aldrich) was used to select the infected cells.

### *In vitro* ubiquitination assay

The recombinant GST-SET/TAF-Iβ, GST-MIB1, GST-RING1B, and GST-H2A were expressed in BL21 (DE3) strain of *Escherichia coli* and purified with Glutathione Sepharose^®^ 4B (Cytiva, Marlborough, MA, USA). For E1 enzyme, recombinant human ubiquitin-activating enzyme E1 (Enzo Life Sciences, Farmingdale, NY, USA) was used. FLAG-ubiquitin was purchased from R&D Systems (Minneapolis, MN, USA). All components were mixed with the reaction buffer (50 mM Tris–HCl [pH 7.5], 2.5 mM MgCl_2_, 2 mM ATP and 2 mM Dithiothreitol [DTT]). Mg-ATP solution (Enzo Life Sciences) was used to activate the E1 enzyme. The reaction was carried out at 37°C for 4 h and stopped by adding sodium dodecyl sulfate (SDS) sample buffer and boiling. Staining with Coomassie brilliant blue (CBB) staining solution (Enzynomics, Daejeon, Korea) and immunoblotting was performed with reaction mixtures after sodium dodecyl sulfate-polyacrylamide gel electrophoresis (SDS-PAGE).

### Complex-immunoprecipitation (co-IP) assay

Harvested cells were trypsinized and lysed in NP-40 lysis buffer (50 mM Tris–HCl [pH 8.0], 200 mM NaCl, 0.5% NP-40, and 1× protease inhibitor cocktail [PIC]) followed by moderate sonication. Lysates were immunoprecipitated with monoclonal anti-FLAG^®^ M2 antibody (F3165, Sigma-Aldrich) with rotation at 4°C for overnight. Then, protein A/G agarose beads (GenDEPOT, Katy, TX, USA) were added to lysates, and the mixture was rotated for 2 h at 4°C. The beads were washed with washing buffer (20 mM Tris–HCl [pH 7.5], 150 mM NaCl, 1 mM EDTA, 1 mM EGTA, 1 mM PMSF, 1× PIC, and 1% Triton X-100) three times. After washing, the supernatants were removed and SDS sample buffer was added and boiled. The bound proteins were analyzed by western blotting with appropriate antibodies.

### Histone extraction

To extract histones, cell pellets were lysed in lysis buffer (0.5% Triton X-100, 2 mM PMSF and 1× PIC) with rotation at 4°C for 30 min. After centrifugation (10 000 g, 10 min), pellets were lysed in 0.4 N HCl, and the mixture was rotated gently at 4°C for overnight. After centrifugation (16 000 g, 10 min), the supernatants were moved to fresh tubes containing 100% TCA, and the tubes were rotated at 4°C for 2 h. White histone pellets were centrifuged at 16 000 g for 10 min and washed with acetone for 2 times. The pellets were resolved in distilled water and used for western blotting.

### Western blotting and antibodies

Harvested cells were incubated in RIPA buffer (50 mM Tris–HCl [pH 8.0], 150 mM NaCl, 0.1% SDS, 0.5% SDC, 1% NP-40, 1× PIC and 1 mM EDTA [pH 8.0]) with gentle agitation at 4°C for 1 h. Cell lysates were clarified by centrifugation at 18 000 g for 15 min at 4°C. After SDS-PAGE, western blotting was performed using the following antibodies: SET/TAF-Iβ (sc-133138, 1:1000), MIB1 (sc-393551, 1:1000), p21 (sc-397, 1:1000), GFP (sc-9996, 1:1000), CBX8 (sc-374332, 1:1000) and β-actin (sc-47778, 1:1000) from Santa Cruz Biotechnology (Dallas, TX, USA); H2AK119ub (8240S, 1:5000), H3K4me3 (9751S, 1:5000) and H3K27me3 (9733S, 1:5000) from Cell Signaling Technology (Danvers, MA, USA); FLAG (F3165, 1:10 000) from Sigma-Aldrich; H3K36me1 (07-548, 1:5000), H3K36me3 (07-549, 1:5000), H3K4me2 (07-030, 1:5000), H3K9me2 (07-441, 1:5000), H3K9me3 (17–625, 1:5000), H2BK120ub (17-650, 1:5000), HA (05-904, 1:5000) and H3 (05-499, 1:5000) from Merck (Rahway, NJ, USA); RAD51 (GTX70230, 1:2500) from GeneTex (Irvine, CA, USA); H3K79me1 (ab2886, 1:5000), H3K79me2 (ab3594, 1:5000), H3K79me3 (ab2621, 1:5000) and H3K36me2 (ab9049, 1:5000) from Abcam (Cambridge, UK). β-actin (ACTIN) and histone H3 were used as loading controls.

### Tissue array and immunohistochemistry (IHC)

Colon carcinoma tissue array slides (CO243a) including colon carcinoma tissues and matched adjacent normal colon tissues were purchased from US Biomax (Derwood, MD, USA). These slides contain formalin-fixed, paraffin-embedded normal colon tissues and colon cancer tissues. First, slides were deparaffinized in xylene and rehydrated in graded ethanol. Antigens were retrieved by heating with microwave oven in 0.01 M citrate buffer (pH 6.0). Endogenous peroxidase activity was quenched by incubating in 3% hydrogen peroxide solution for 6 min. Next, slides were incubated with normal goat serum for 30 min at room temperature for blocking. Subsequently, the slides were incubated with primary antibody against SET/TAF-Iβ (1:500, ab1183 from Abcam) for 2 h at room temperature followed by incubation with biotinylated anti-rabbit secondary antibody and streptavidin-horseradish peroxidase (Zymed Laboratories, South San Francisco, CA, USA). DAB substrate kit (SK-4100, Vector Laboratories, Newark, CA, USA) was used as a chromogen and Eosin B (Sigma-Aldrich) was used for counterstaining.

### RNA sequencing (RNA-seq)

For RNA-seq, 4 samples (HCT116 shNC#1, shNC#2, shMIB1#1, shMIB1#2) were prepared in duplicates. RNA sequencing was performed by Theragen Bio Inc. (Seongnam, Korea). Libraries were prepared for 150-bp paired-end sequencing using the TruSeq Stranded mRNA Sample Prep Kit (Illumina, San Diego, CA, USA). The mRNA molecules were purified and fragmented from 0.1 to 1 μg of total RNA using oligo (dT) magnetic beads. Fragmented mRNAs were synthesized as single-stranded complementary DNAs (cDNAs) through random hexamer priming. Double-stranded cDNA was prepared by using this cDNA as a template for second strand synthesis. After a sequential process of end repair, A-tailing, and adapter ligation, cDNA libraries were amplified using PCR. The quality of these cDNA libraries was evaluated using the Agilent 2100 BioAnalyzer (Agilent Technologies, Santa Clara, CA, USA). They were quantified using the KAPA Library Quantification Kit (Kapa Biosystems, Wilmington, MA, USA) according to the manufacturer's library quantification protocol. Following cluster amplification of denatured templates, sequencing was performed using paired-end (2 × 150 bp) reads using Illumina NovaSeq 6000 (Illumina). Low-quality reads were filtered based on the following criteria: reads containing more than 10% of skipped bases (marked as ‘N’s), reads containing more than 40% of bases with quality scores of less than 20, and reads with average quality scores for each read of less than 20. The filtering process was performed using in-house scripts. Filtered reads were mapped to the reference genome (hg38) related to the species using the aligner STAR v.2.4.0b. Gene expression levels were measured using Cufflinks (v2.1.1) and the gene annotation database of the species. For differentially expressed gene (DEG) analysis, gene-level count data were generated using HTSeq-count v0.6.1p1. Based on the calculated read count data, DEGs were identified using the R package ‘TCC’. The normalization factors were calculated using the iterative DEGES/edgeR method. Based on DEG analysis results, gene ontology (GO) analysis was performed using DAVID 2021 (17,18) or ShinyGO (v0.76) (19). To visualize the GO analysis results, REVIGO (20) was used. All plots and visualizations were generated using R 4.1.2 (https://www.r-project.org/). Gene set enrichment analysis (GSEA) was performed using GSEA software (v4.1.0) (21,22).

### Reverse transcription followed by quantitative PCR (RT-qPCR)

Total RNA was extracted from harvested cells using the Tri-RNA reagent (Favorgen, Pingtung, Taiwan) according to the manufacturer's protocol. The cDNA was synthesized from 1 μg of RNA. The RNA was incubated with oligo (dT) primers (Invitrogen, Waltham, MA, USA) at 70°C for 5 min and M-MLV reverse transcriptase (Enzynomics) and dNTP (Enzynomics) were then added. Synthesis was performed by incubation at 42°C for 60 min followed by enzyme inactivation at 95°C. SYBR^®^ Green Supermix Kit (Takara Bio, Kusatsu, Japan) was used for RT-qPCR. Total 39 cycles of amplification were performed. Each cycle consisted of denaturation at 94°C, annealing at primer-specific temperatures, and extension at 72°C. The mean threshold cycle (Ct) and standard error were calculated using the Ct value of each sample. The normalized mean Ct value (ΔCt) was calculated by subtracting the mean Ct value of *ACTB* from that of the target gene. The ΔΔCt value was calculated as the difference between the control ΔCt and the ΔCt values for each sample. Finally, the n-fold change in mRNA levels was calculated as 2^−ΔΔCt^. The following primer sets (forward, reverse and 5′ to 3′) were used for RT-qPCR: *MIB1* (AGTTGGCTTTGAGGGCATGT, TGCCATTCTGCTCACCTAGC), *SET/TAF-Iβ* (CATCTGAATGAGAGTGGTGATCC, TCTCTGGTTCCTCATGCTGCCT), *YES1* (GAGAATCTTTGCGACTAGAGG, CTGGCATCATTGTACCTGG), *RAD51* (TTTGGAGAATTCCGAACTGG, CATCACTGCCAGAGAGACCA), *BRCA1* (TCTGAAGACAGAGCCCCAGA, GCTTCTCCCTGCTCACACTT), *LAMC2* (CCAGAAGGTTTCAGATGCC, TGGCTTCCAAGTTCAGACTC), *OAS1* (ATTGACAGTGCTGTTAACATCATC, AGATCAATGAGCCCTGCATAAACC), *DPP4* (AAGATGGAACTGCTTAGTGG, AGAGCTTCTATCCCGATGAC), and *ACTB* (TCCCTGGAGAAGAGCTACGA, AGGAAGGAAGGCTGGAAGAG).

### Chromatin immunoprecipitation quantitative real-time PCR (ChIP-qPCR)

ChIP-qPCR assay was performed as previously described (23). Briefly, cells were cross-linked with 1% formaldehyde for 10 min and 125 mM glycine was added and incubated for 5 min at room temperature. Cells were harvested by cell scraper and lysed in SDS lysis buffer (1% SDS, 10 mM EDTA, 50 mM Tris-HCl [pH 8.1] and 2× PIC). Next, cells were sonicated and clarified lysates were subjected to immunoprecipitation reaction with indicated antibodies (8240S for H2AK119ub and 9733S for H3K27me3 from Cell Signaling Technology; 06-598 for H4-Ac and 12-370 for normal rabbit IgG from Merck). Eluted immunoprecipitants were reverse cross-linked. Purified DNA fragments were amplified by PCR using specific primers. The mean threshold cycle (Ct) and standard error values were calculated from individual Ct values, obtained from duplicate reactions per stage. The normalized mean Ct value was estimated as ΔCt by subtracting the mean Ct value of the input from that of each samples. The following primer sets targeting promoter region of each gene (forward, reverse and 5′ to 3′) were used for ChIP-qPCR: *RAD51* (TCTTCTCGAGCTTCCTCAGC, AGCGCTCTTGTGGTTTGTTT), *YES1* (TGATGAGGGTGTGAGTAACGC, TGGTTGGCTGTTTGCTTCTC), *BTG2* (CCCGGCTACACTGTATATTGACTTGG, GGGTTTCATCACGTTGGTCAGGAT), and *CDKN1A* (GTGGCTCTGATTGGCTTTCTG, CTGAAAACAGGCAGCCCAAG)

### In-gel protein digestion and liquid chromatography with tandem mass spectrometry (LC–MS/MS) with nano LC-LTQ-orbitrap elite analysis

Mass spectrometry and proteomic analyses were carried out at the Korea Basic Science Institute (KBSI; Daejeon, Korea) as previously described (24). Briefly, FLAG-SET/TAF-Iβ was overexpressed in HCT116 cells and immunoprecipitated with anti-FLAG antibodies (F3165, Sigma-Aldrich). The immunoprecipitates were separated by SDS-PAGE. The gel was stained with CBB staining solution (Enzynomics). The stained gel was sliced into 10 pieces and subjected to in-gel digestion with sequencing-grade modified trypsin (Promega, Madison, WI, USA). The digested peptides were analyzed using reversed-phase capillary high-performance liquid chromatography (HPLC) directly coupled to a Finnigan LCQ ion trap mass spectrometer (Thermo Fisher Scientific, Waltham, MA, USA). All tryptic peptides were recovered by the extracting process followed by concentrating process with a vacuum centrifuge. Digested plasma samples were analyzed with LC–MS/MS system including a nanoAcquity UPLC system (Waters, Milford, MA, USA) and an LTQ Orbitrap Elite mass spectrometer (Thermo Fisher Scientific).

### Chromatin immunoprecipitation sequencing (ChIP-seq) analysis

ChIP-seq data of anti-H2AK119ub and anti-RING1B in leukemia cells were obtained from GSE54580 (25). ChIP-seq data with anti-SET/TAF-Iβ in leukemia cells were acquired from unpublished data in our laboratory. Custom tracks were plotted by pyGenomeTrack (26) with each bigWig file.

### Cell viability and proliferation assay

Cells were seeded in 48-well plates at density of 5 × 10^3^ cells per well. For cell viability assay, 3-(4,5-dimethylthiazol-2-yl)-2,5-diphenyltetrazolium bromide (MTT) (Glentham Life Sciences, Corsham, Wiltshire, UK) was added to the cells after 24 h at a final concentration of 0.1 mg/ml and cells were incubated for 4 h at 37°C. After 4 h, medium including MTT was removed and DMSO was added. The absorbance at 575 nm was measured with Epoch™ Microplate Spectrophotometer (BioTek, Winooski, VT, USA). For colony formation assay, cells were seeded in the same way and stained with 0.01% crystal violet after MeOH fixation. Colonies were counted with Image J software.

### Cell cycle analysis and BrdU incorporation assay

Cells were harvested by trypsinization and washed with PBS. Cells were resuspended in PBS and cold EtOH was added dropwise to final concentration of 70%, and the mixture was incubated overnight with gentle agitation at 4°C. Cells were washed with PBS and resuspended in propidium iodide (PI, Invitrogen) staining PBS buffer (50 μg/ml PI, 100 μg/ml RNase A, 0.1% Triton X-100) and incubated overnight at 4°C in dark. For 5′-bromo-2′-deoxyuridine (BrdU) incorporation assay, cells were incubated with 10 μM BrdU (Sigma-Aldrich) for 30 min before trypsinization. Harvested cells were fixed with EtOH in the same way. Cells were washed with PBS and incubated with 2 N HCl for 20 min at room temperature. After washing with PBS, cells were incubated with 0.1 M Na_2_B_4_O_7_ for 10 min at room temperature. Cells were washed with staining PBS buffer and stained with APC-BrdU (17-5071-42, Invitrogen) and 7-amino-actinomycin D (7-AAD) (BioLegend, San Diego, CA, USA) overnight at 4°C. Fluorescence-activated cell sorting (FACS) analysis was performed with Attune™ NxT Flow Cytometer (Invitrogen).

### Statistical analyses

Appropriate two-tailed Student's *t*-test was used for two-group comparisons. The one-way analysis of variance model (ANOVA) followed by appropriate multiple comparisons test was performed for more than three-group comparisons. The statistical analyses and visualization were performed using GraphPad Prism (GraphPad Software, San Diego, CA, USA). Quantifications of wester blotting and IHC were performed with Image J software (27). The mean and standard deviation (SD) or standard error of the mean (SEM) are shown in bar graphs and information about replicates are described in figure legends.

## RESULTS

### SET/TAF-Iβ is upregulated in colon cancer

**Figure 1.**
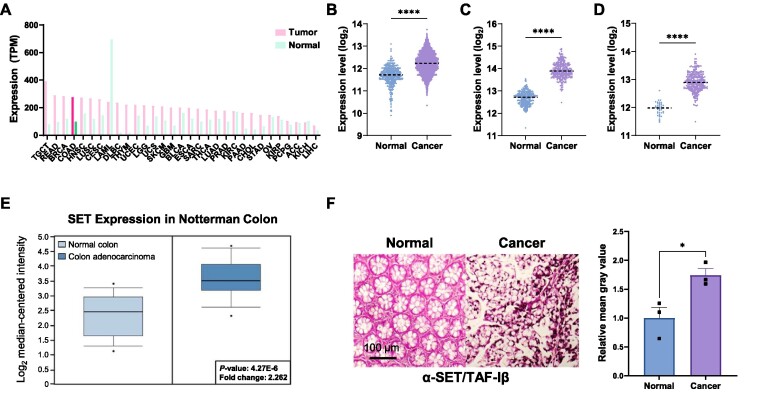
SET/TAF-Iβ is overexpressed in colon cancer. (**A**) Expression pattern of SET/TAF-Iβ across 31 tumor types. The y-axis indicates expression levels in transcripts per million (TPM). (**B**) Expression analysis of SET/TAF-Iβ in normal (*n* = 397) and cancerous (*n* = 3775) colon tissues using the data available in the GENT2 database. The dotted lines indicate median values. The *P*-value was calculated with unpaired Student's *t*-test with Welch's correction. The dotted line indicates the mean value. ****P < 0.0001. (**C**) Expression analysis of SET/TAF-Iβ in tissues from healthy donors (n = 304) tissues and colon cancer patients (*n* = 290) using the available in the TCGA TARGET GTEx database. The dotted lines indicate the median values. The *P*-value was calculated with unpaired Student's *t*-test with Welch's correction. The dotted line indicates the mean value. ****P< 0.0001. (**D**) Expression analysis of SET/TAF-Iβ in tissues from healthy donors (*n* = 41) and colon cancer patients (*n* = 286) using the data available in the TCGA Colon Cancer (COAD) database. The dotted lines indicate the median values. The *P*-value was calculated with unpaired Student's *t*-test with Welch's correction. The dotted line indicates the mean value. ****P < 0.0001. (**E**) SET/TAF-Iβ expression analysis of the data in Notterman Colon in Oncomine database. The boxes extend from the 25th to the 75th percentiles and the line in the middle of the box indicates the median value. The *P*-value was calculated with unpaired Student's *t*-test. (**F**) Immunohistochemistry (IHC) staining result of colon cancer tissue array. Tissues were immunostained with anti-SET/TAF-Iβ antibody (left). IHC result was quantified by mean gray value (right). Scale bar = 100 μm. The *P*-value was calculated with unpaired Student's *t*-test. Data are expressed as mean ± SEM (*n* = 3). *P < 0.05.

Expression level of SET/TAF-Iβ is upregulated in multiple tumor tissues (28). We examined the expression patterns of SET/TAF-Iβ in colon cancer tissues by analyzing the publicly available datasets from several databases. Colon cancer tissues, which showed upregulated SET/TAF-Iβ expression, exhibited the 4^th^ highest level of SET/TAF-Iβ expression among the 31 tumor types assessed (29) (Figure 1A). Gene Expression database of Normal and Tumor tissues (GENT2) (30) database analysis also indicated that the expression levels of SET/TAF-Iβ in colon cancer tissues were greater than those in normal colon tissues (Figure 1B). In addition, analyses of data from the Cancer Genome Atlas (TCGA) and Genotype-Tissue Expression (GTEx) revealed that gene expression levels of SET/TAF-Iβ were upregulated in colon adenocarcinoma tumor samples (Figure 1C), and similar results were obtained by the analysis of data from TCGA colon cancer database (31) (Figure 1D). Moreover, SET/TAF-Iβ exhibited about 2-fold higher expression levels in colon adenocarcinoma according to Notterman colon data from Oncomine (32) (Figure 1E). Consistent with these, immunohistochemistry (IHC) staining using tissue microarray revealed that SET/TAF-Iβ was highly upregulated in malignant colon tumor tissues compared with the adjacent normal colon tissues (Figure 1F). Taken together, these results suggest that the expression of SET/TAF-Iβ is upregulated in colon cancer.

### SET/TAF-Iβ regulates mono-ubiquitination of histone H2A at K119 in HCT116 cells

**Figure 2.**
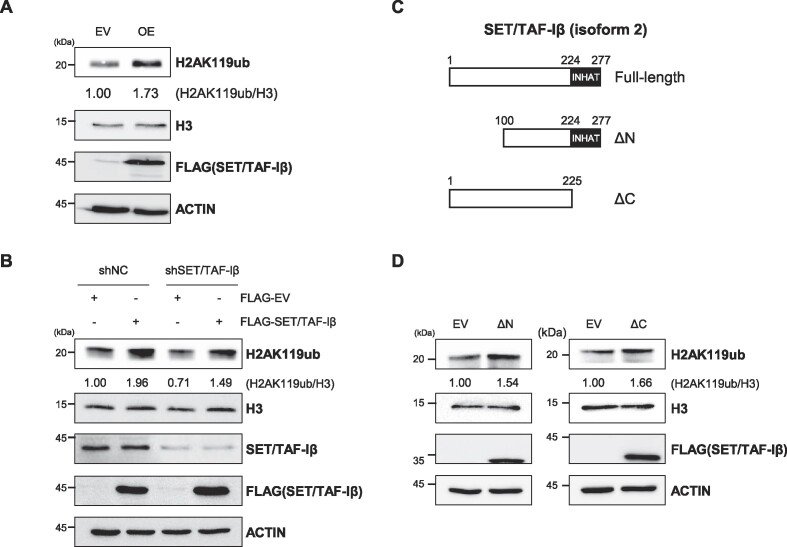
Overexpression of SET/TAF-Iβ promotes H2AK119ub in HCT116 cells. (**A**) Western blot analysis of HCT116 cells overexpressing SET/TAF-Iβ. Cells were transfected with FLAG-SET/TAF-Iβ plasmid. EV, empty vector; OE, overexpression. (**B**) Western blot analysis of SET/TAF-Iβ-recovered HCT116 cells. The control (shNC) and SET/TAF-Iβ-knockdown cells were transfected with EV or SET/TAF-Iβ plasmid. (**C**) Domain maps of SET/TAF-Iβ constructs. INHAT domain indicates inhibitor of acetyltransferases domain. (**D**) Western blot analysis of HCT116 cells overexpressing partial constructs of SET/TAF-Iβ indicated in (C).

Next, we asked whether increased SET/TAF-Iβ expression could affect histone modifications in HCT116 cells. Among the several candidate modifications we tested, mono-ubiquitination of histone H2A lysine 119 (H2AK119ub) was upregulated upon SET/TAF-Iβ overexpression in HCT116 cells (Figure 2A and Supplementary Figure S1A). Induction of H2AK119ub was also observed in other cell lines including H1299, MCF-7, HeLa and HEK293T (Supplementary Figure S2B–E). In addition, we overexpressed SET/TAF-Iβ in SET/TAF-Iβ-knockdown cells and analyzed the levels of H2AK119ub. Intriguingly, the decrease in H2AK119ub levels were restored, resulting in H2AK119ub levels similar to those in control cells (Figure 2B). These findings suggest that SET/TAF-Iβ have a role in the regulation of H2AK119ub levels in HCT116 cells.

SET/TAF-Iβ has a highly acidic inhibitor of acetyltransferases (INHAT) domain at its C-terminal, which acts as an inhibitor of histone acetylation (1). To find out whether INHAT domain is necessary for SET/TAF-Iβ-mediated increase in H2AK119ub levels in HCT116 cells, we used constructs of SET/TAF-Iβ with or without the INHAT domain (Figure 2C). When SET/TAF-Iβ construct containing the INHAT domain (ΔN) was overexpressed, H2AK119ub abundance increased to levels comparable to those observed in cells overexpressing the full-length SET/TAF-Iβ (Figure 2D). In cells overexpressing the construct lacking the INHAT domain (ΔC), increased levels of H2AK119ub were still detected (Figure 2D) indicating that INHAT domain is dispensable for regulating H2AK119ub in HCT116 cells. Taken together, these results suggest that SET/TAF-Iβ promotes H2AK119ub, and this regulatory mechanism is independent of the INHAT domain of SET/TAF-Iβ.

### SET/TAF-Iβ interacts with E3 ligase MIB1 to promote histone H2A mono-ubiquitination at K119

**Figure 3.**
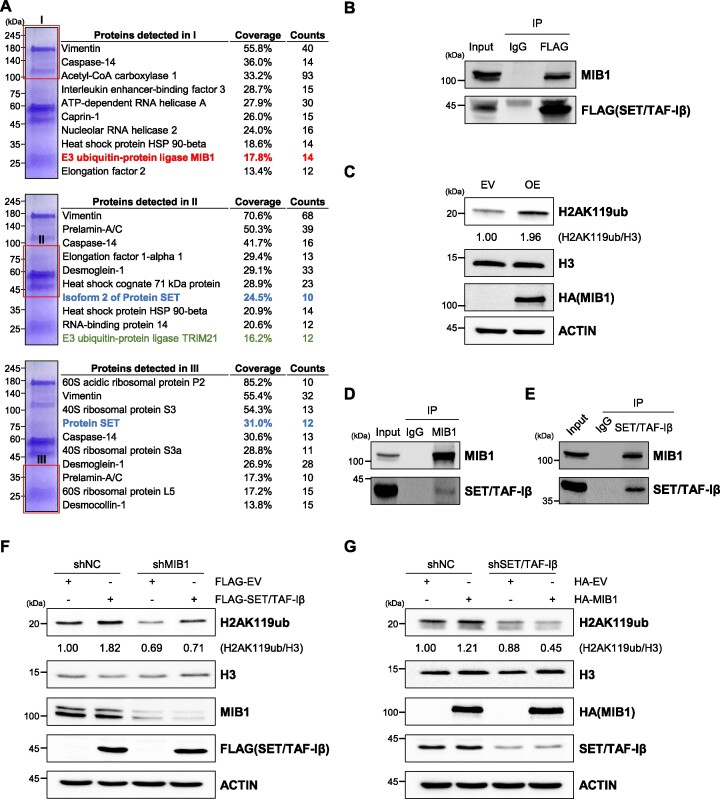
SET/TAF-Iβ interacts with E3 ligase MIB1 to mediate mono-ubiquitination of H2AK119. (**A**) Liquid chromatography with tandem mass spectrometry (LC–MS/MS) analysis of immunoprecipitates obtained with FLAG antibody from HCT116 cells overexpressing FLAG-SET/TAF-Iβ. SET/TAF-Iβ and E3 ligase TRIM21, MIB1 are colored in blue, green and red, respectively. (**B**) Western blot after immunoprecipitation (IP) to validate LC–MS/MS analysis results. HCT116 cells overexpressing FLAG-SET/TAF-Iβ were subjected to IP with antibody targeting FLAG tag. (**C**) Western blot analysis of HCT116 cells overexpressing MIB1. (**D**) Complex-immunoprecipitation (Co-IP) assay with MIB1 antibody to assess the endogenous interaction between MIB1 and SET/TAF-Iβ. (**E**) Co-IP assay with SET/TAF-Iβ antibody to assess the endogenous interaction between MIB1 and SET/TAF-Iβ. (**F**) Western blot analysis of MIB1 knockdown HCT116 cells. Control (shNC) and MIB1 knockdown (shMIB1) cells were transfected with EV or SET/TAF-Iβ. (**G**) Western blot analysis of MIB1 knockdown HCT116 cells. Control (shNC) and SET/TAF-Iβ knockdown (shSET/TAF-Iβ) cells were transfected with EV or MIB1.

Given that SET/TAF-Iβ is upregulated in colon cancer and induces H2AK119ub in HCT116 cells, we next attempted to identify its binding partners that might, along with SET/TAF-Iβ, be responsible for the regulation of H2AK119ub levels. Immunoprecipitation (IP)-mass spectrometry (MS) analysis of HCT116 cells overexpressing FLAG-SET/TAF-Iβ indicated that five ubiquitin ligase (E3) enzymes, along with other proteins, interacted with SET/TAF-Iβ (Figure 3A). Among them, we focused on E3 ligases MIB1 and TRIM21 as possible candidates involved in H2AK119ub modification as they showed higher coverages and counts in proteomic analysis (Figure 3A). The result of MS analysis was verified by IP with HCT116 cells overexpressing FLAG-SET/TAF-Iβ (Figure 3B). We first overexpressed TRIM21 and found out that H2AK119ub level did not change in HCT116 cells (Supplementary Figure S2A). On the other hand, when we overexpressed MIB1 in HCT116 cells, H2AK119ub level remarkably increased (Figure 3C). To rule out the binding of FLAG antibody to MIB1, we tested whether MIB1 is detected by FLAG-empty vector overexpression followed by α-FLAG IP. We found that FLAG antibody does not bind to endogenous MIB1 in HCT116 cells (Supplementary Figure S2B). Next, we examined the endogenous interaction between MIB1 and SET/TAF-Iβ in HCT116 cells by IP. As a result, we were able to detect endogenous interactions between SET/TAF-Iβ and MIB1, reciprocally (Figure 3D and E). In addition, in HCT116 and 293T cells co-transfected with HA-MIB1 and FLAG-SET/TAF-Iβ, we detected the interaction between MIB1 and SET/TAF-Iβ including partial constructs of SET/TAF-Iβ (Supplementary Figure S2C and D).

To pinpoint the exact role of SET/TAF-Iβ in MIB1-mediated H2AK119ub, we overexpressed SET/TAF-Iβ in MIB1-knockdown HCT116 cells and found that there was no increase in H2AK119ub levels under these conditions (Figure 3F). MIB1 overexpression in SET/TAF-Iβ-knockdown cells did not alter H2AK119ub levels, either (Figure 3G). Notably, when SET/TAF-Iβ and MIB1 were overexpressed simultaneously, they showed a synergistic effect in upregulating H2AK119ub levels in HCT116 cells (Supplementary Figure S2E). These observations strongly indicate a potential role of SET/TAF-Iβ as an E2-conjugating enzyme working with E3 ligase MIB1. As expected, various histones including H2A were identified as SET/TAF-Iβ-interacting proteins (Supplementary Figure S2F). Therefore, our results suggest that SET/TAF-Iβ and E3 ligase MIB1 work together to promote H2AK119ub in HCT116 cells.

### MIB1 and SET/TAF-Iβ ubiquitinates histone H2A at K119 *in vitro*

**Figure 4.**
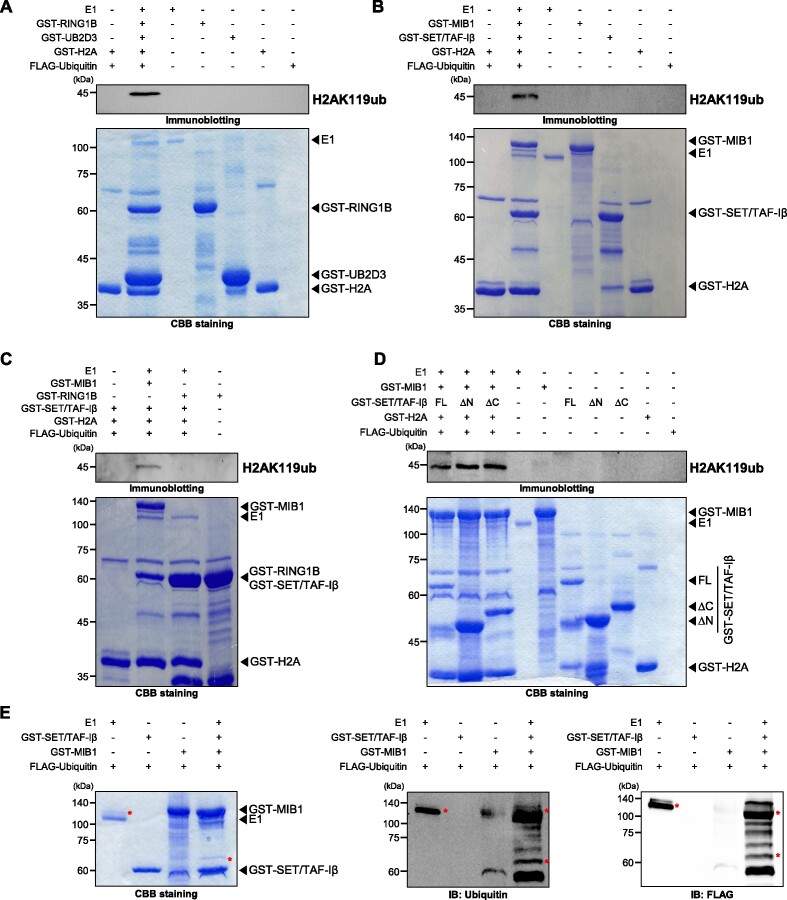
SET/TAF-Iβ and MIB1 catalyze mono-ubiquitination of H2AK119 *in vitro*. (**A**) *In vitro* ubiquitination assay was performed with purified GST-RING1B and GST-UB2D3. GST-H2AK119ub was immunoblotted with anti-H2AK119ub antibody. (**B**) *In vitro* ubiquitination assay was performed with purified GST-MIB1 and GST-SET/TAF-Iβ. GST-H2AK119ub was immunoblotted with anti-H2AK119ub antibody. (**C**) *In vitro* ubiquitination assay with GST-RING1B and GST-SET/TAF-Iβ. GST-H2AK119ub was immunoblotted with anti-H2AK119ub antibody. (**D**) *In vitro* ubiquitination assay with GST-MIB1 and GST-SET/TAF-Iβ partial constructs. GST-H2AK119ub was immunoblotted with anti-H2AK119ub antibody. FL, full-length. (**E**) *In vitro* ubiquitination assay without substrate protein, GST-H2A. Ubiquitin-attached E1 and GST-SET/TAF-Iβ were immunoblotted with anti-ubiquitin or anti-FLAG antibodies.

Since we found that H2AK119ub is regulated by MIB1 and SET/TAF-Iβ in HCT116 cells, we next performed an *in vitro* ubiquitination assay. His-tagged recombinant ubiquitin activating enzyme E1, purified GST-MIB1, GST-SET/TAF-Iβ and GST-H2A were used in the ubiquitination assay. First, we used GST-RING1B and GST-UB2D3 to check whether our *in vitro* ubiquitination assay is capable of mediating H2AK119ub since RING1B is the most well-known E3 ligase responsible for H2AK119ub (33). As a result, we were able to detect a specific band around 45 kDa using H2AK119ub-specific antibody (Figure 4A). Considering the molecular weight of GST-H2A and FLAG-ubiquitin, we conclude that 45 kDa is the proper size of GST-H2AK119ub. When ubiquitination reaction was induced with GST-MIB1 and GST-SET/TAF-Iβ, we were also able to detect a specific band around 45 kDa using the same antibody (Figure 4B). On the other hand, when each component of the reaction was used separately in the ubiquitination assay, the specific band at 45 kDa was not detected (Figure 4A and B). These results suggest that SET/TAF-Iβ acts as E2-conjugating enzyme along with E3 ligase MIB1 and accommodate ubiquitination reaction toward histone H2A at K119 *in vitro*.

Intriguingly, when GST-RING1B was used instead of GST-MIB1, the specific GST-H2AK119ub band at 45 kDa was not detected (Figure 4C). In fact, ectopic expression followed by co-IP analyses indicate that SET/TAF-Iβ and RING1B did not interact each other (Supplementary Figure S3A). Furthermore, when we tested interactions between SET/TAF-Iβ and other polycomb repressive complex 1 (PRC1)-associated proteins both canonical and noncanonical including KDM2B, BMI1, RYBP and CBX8, SET/TAF-Iβ did not interact with any of them (Supplementary Figure S3B–E). In addition, overexpression and knockdown of SET/TAF-Iβ or MIB1 did not change protein level of RING1B in HCT116 cells (Supplementary Figure S3F–H). These results strongly suggest that SET/TAF-Iβ and MIB1 mediate PRC1-independent H2AK119ub.

When partial constructs of SET/TAF-Iβ (Figure 2C) were used in the ubiquitination assay, all constructs were able to induce H2AK119ub with MIB1 (Figure 4D). Previously, we checked that overexpression of SET/TAF-Iβ partial constructs induced H2AK119ub in HCT116 cells (Figure 2D). Also, these constructs were able to interact with MIB1 in HCT116 cells (Supplementary Figure S2D). These results consistently suggest that N-terminal domain or INHAT domain is dispensable for inducing ubiquitination reaction.

In addition, we tested whether SET/TAF-Iβ is capable of possessing ubiquitin during ubiquitination reaction by performing *in vitro* ubiquitination assay without substrate GST-H2A. In ubiquitination reaction, ubiquitin is activated by ubiquitin-activating enzyme (E1) and activated ubiquitin is transferred to ubiquitin-conjugating enzyme (E2), finally, ubiquitin-conjugated E2 interacts with ubiquitin ligase enzyme (E3) to mediate ubiquitination of target substrate protein (34). We hypothesized that if *in vitro* ubiquitination assay is performed without the substrate protein, then the activated ubiquitin will remain attached to E1 or E2 enzymes because there is no substrate protein which ubiquitin should be transferred to. As a result, we found shifted E1 and GST-SET/TAF-Iβ bands due to size of the ubiquitin indicating ubiquitin is attached to both proteins (Figure 4E, left panel, asterisks). Also, the shifted GST-SET/TAF-Iβ band was not detected when E1 enzyme was absent. In addition, immunoblotting with anti-ubiquitin and anti-FLAG antibodies showed that the shifted bands were detectable with antibodies against ubiquitin or FLAG indicating that the shifted bands represent ubiquitin-attached E1 or SET/TAF-Iβ (Figure 4E, right panel, asterisks).

Altogether, these data suggest that E3 ligase MIB1 directly and specifically catalyzes the H2AK119ub through E2-conjugating activity of SET/TAF-Iβ in PRC1-independent mechanism.

### MIB1 regulates DNA replication-related genes in HCT116 cells

**Figure 5.**
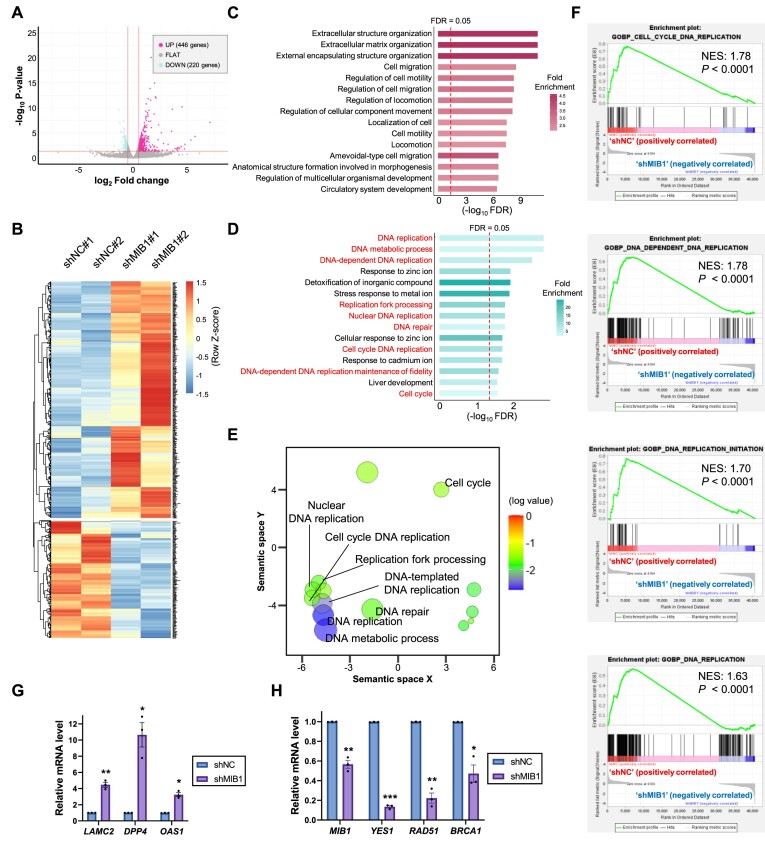
MIB1 regulates expression of genes related to DNA replication in HCT116 cells. (**A**) Volcano plot showing differentially expressed genes (DEGs) in MIB1-depleted cells. Upregulated and downregulated DEGs were colored in magenta and cyan, respectively. The red lines indicate threshold for each value (|log_2_ fold change| $ \ge$ 0.5 and *P*-value $ \le$ 0.05). (**B**) Heatmap showing DEGs in (A). (**C**) Gene ontology (GO) analysis result with upregulated DEGs in (B). The x-axis indicates -log_10_ value of false discovery rate (FDR). The red dotted-line indicates FDR value of 0.05. (**D**) Results of GO analysis of downregulated DEGs in (B). (**E**) Reduce + Visualize Gene Ontology (REVIGO) plot of GO analysis results in (D). Size of circle indicates log size of each terms calculated by REVIGO. (**F**) Gene Set Enrichment Analysis (GSEA) of RNA-seq data. NES, normalized enrichment score. (**G**) Reverse transcription followed by quantitative PCR (RT-qPCR) analysis of identified upregulated target genes in MIB1-deficient HCT116. The *P*-values were calculated with paired Student's *t*-test. Data are expressed as mean ± SEM (*n* = 3). **P < 0.01 and *P < 0.05. (**H**) RT-qPCR analysis of identified downregulated target genes in MIB1-deficient HCT116 cells. The *P*-values were calculated with paired Student's *t*-test. Data are expressed as mean ± SEM (*n* = 3). ***P < 0.001, **P < 0.01 and *P < 0.05.

Since we found that the histone mark H2AK119ub is regulated by MIB1 in HCT116 cells, we performed RNA-seq analysis to find out genes regulated by MIB1 in HCT116 cells. We depleted MIB1 expression in HCT116 cells and analyzed the resultant transcriptome. Overall, 446 genes were upregulated, and 220 genes were downregulated upon MIB1 depletion (Figure 5A and B). Since H2AK119ub is a well-known repressive marker for transcription (35), it seems reasonable that twice more genes are upregulated in response to inhibition of MIB1 and H2AK119ub. Next, we performed Gene Ontology (GO) analysis of differentially expressed genes (DEGs). When the upregulated DEGs were subjected to GO analysis, GO terms related to extracellular structure organization were identified as the top hits (Figure 5C).

Interestingly, when downregulated genes were used as input for GO analysis, GO terms related to DNA replication and DNA repair showed the highest enrichment levels (Figure 5D). When these GO terms were visualized through REVIGO (20), GO terms related to DNA replication were clustered into similar positions (Figure 5E). Moreover, when we performed Gene Set Enrichment Analysis (GSEA) with RNA-seq data, gene sets of biological processes including cell cycle DNA replication, DNA-dependent DNA replication, DNA replication initiation, and DNA replication were negatively correlated with MIB1 depletion (Figure 5F). The RNA-seq results for some of the identified genes were validated by reverse transcription followed by quantitative PCR (RT-qPCR). The mRNA levels of *LAMC2*, *DPP4* and *OAS1*, which showed log_2_ fold changes of 1.97, 1.91 and 3.54 in RNA-seq, respectively, were higher in MIB1-depleted cells (Figure 5G). On the other hand, the expression levels of downregulated target genes *YES1*, *RAD51* and *BRCA1*, which showed log_2_ fold changes of –1.40, –1.13 and –0.52, respectively, decreased upon *MIB1* depletion (Figure 5H). YES1 has been proposed to regulate cell cycle progression by phosphorylating the cyclin-dependent kinase 4 (CDK4) (36). RAD51 and BRCA1 are also known to regulate meiotic and mitotic cell cycle in addition to DNA repair process (37,38). These results suggest that MIB1 positively regulates genes related to DNA replication in HCT116 cells.

### SET/TAF-Iβ regulates H2AK119ub and H3K27me3 abundance in promoter of target genes and shows nonoverlapping occupancy with RING1B

**Figure 6.**
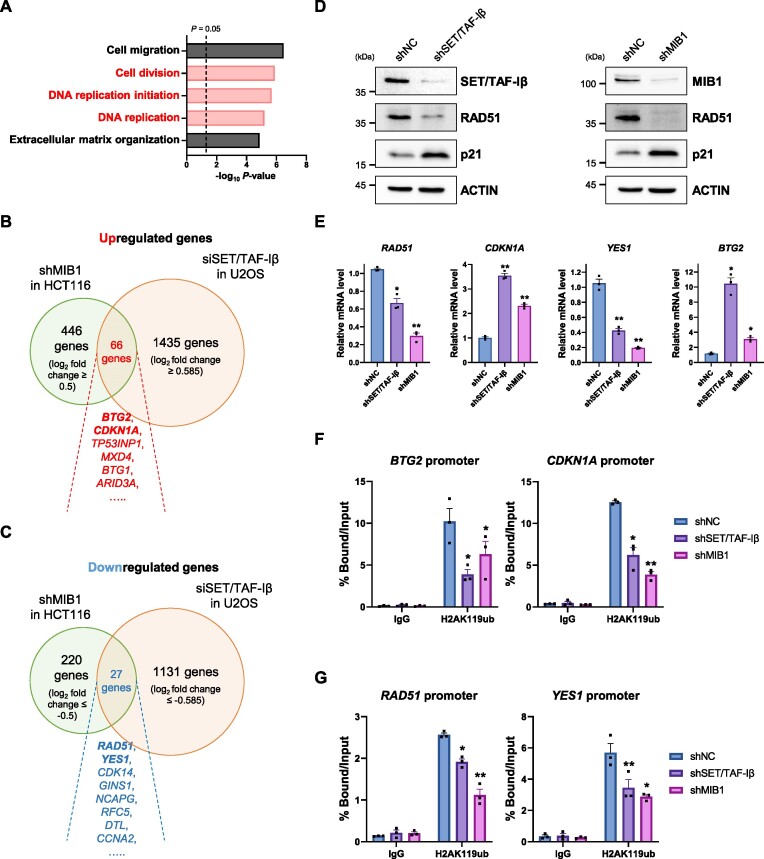
SET/TAF-Iβ and MIB1 regulate H2AK119ub at their target gene promoters. (**A**) GO analysis result with downregulated DEGs in SET/TAF-Iβ-depleted U2OS cells. The dotted-line indicates *P*-value of 0.05. (**B**) Venn diagram showing overlapping upregulated DEGs in shMIB1 HCT116 cells and siSET/TAF-Iβ U2OS cells. (**C**) Venn diagram showing overlapping downregulated DEGs in shMIB1 HCT116 cells and siSET/TAF-Iβ U2OS cells. (**D**) Western blot analysis of SET/TAF-Iβ- or MIB1-depleted HCT116 cells. (**E**) RT-qPCR analysis of overlapping target genes of SET/TAF-Iβ and MIB1 in HCT116 cells. The *P*-values were determined using one-way ANOVA followed by Dunnett's multiple comparisons test. Data are expressed as mean ± SEM (*n* = 3). **P < 0.01 and *P < 0.05. (**F**) Chromatin immunoprecipitation quantitative real-time PCR (ChIP-qPCR) assay to evaluate H2AK119ub level in promoters of upregulated DEGs. Specific primer sets targeting promoter regions of *BTG2* or *CDKN1A* were used. The *P*-values were determined using one-way ANOVA followed by Dunnett's multiple comparisons test. Data are expressed as mean ± SEM (*n* = 3). **P < 0.01 and *P < 0.05. (**G**) ChIP-qPCR assay to evaluate H2AK119ub level in promoters of downregulated DEGs. Specific primer sets targeting promoter regions of *RAD51* or *YES1* were used. The *P*-values were determined using one-way ANOVA followed by Dunnett's multiple comparisons test. Data are expressed as mean ± SEM (*n* = 3). **P < 0.01 and *P < 0.05.

Previous study has reported transcriptome changes induced by depletion of SET/TAF-Iβ in U2OS cells and by analyzing published RNA-seq data, we were able to find out target genes of SET/TAF-Iβ in U2OS cells (7). Importantly, GO analysis with downregulated DEGs revealed that these genes were found out to be related to DNA replication process (Figure 6A). These results indicate that SET/TAF-Iβ might also regulate DNA replication and cell cycle progression in eukaryotic cells.

Next, we searched for overlapping target genes of SET/TAF-Iβ and MIB1. When we compared our RNA-seq data in MIB1-depleted HCT116 cells and RNA-seq data in SET/TAF-Iβ-depleted U2OS cells, we were able to identify 66 upregulated genes and 27 downregulated genes commonly regulated by both proteins (Figure 6B and C). To validate these results, we evaluated protein levels of RAD51 and p21 in shSET/TAF-Iβ and shMIB1 HCT116 cells, respectively. The protein level of p21, which is encoded by *CDKN1A*, increased and expression of RAD51 decreased after depletion of SET/TAF-Iβ or MIB1 in HCT116 cells (Figure 6D). In addition, RT-qPCR analysis revealed that mRNA levels of *RAD51*, *CDKN1A*, *YES1* and *BTG2* were regulated by knockdown of SET/TAF-Iβ or MIB1 in HCT116 cells (Figure 6E). These results suggest that *RAD51*, *CDKN1A*, *YES1* and *BTG2* are target genes of SET/TAF-Iβ and MIB1 in HCT116 cells.

H2AK119ub is well-known to regulate transcription. To evaluate H2AK119ub levels in promoters of the identified target genes, we performed chromatin immunoprecipitation quantitative real-time PCR (ChIP-qPCR) assay. When we checked H2AK119ub levels in promoters of upregulated target genes, H2AK119ub levels remarkably decreased in shSET/TAF-Iβ and shMIB1 cells (Figure 6F). Interestingly, when downregulated genes were subjected to ChIP-qPCR assay, the promoter H2AK119ub level also decreased (Figure 6G). Although PRC1-mediated H2AK119ub is well-known for suppressing transcription (35), the recruitment of non-canonical PRC1 to transcriptionally active genes have been studied (39,40). Recent studies suggest that depletion of PRC1 binding and function downregulated transcription of target genes (41–43). Also, another study revealed that ubiquitinated H2A was strikingly enhanced in transcribed genes compared to non-transcribed satellite chromatin (44). As expected, knockdown of SET/TAF-Iβ increased acetylation of histone H4 at their target genes (Supplementary Figure S4A and B). Intriguingly, knockdown of MIB1 also enhanced H4 acetylation level suggesting a possible role of MIB1 in regulating histone acetylation and transcription. Since H2AK119ub is essential for the H3K27 methylation deposition, we next examined tri-methylated histone H3 lysine 27 (H3K27me3) at same regions. Interestingly, knockdown of SET/TAF-Iβ remarkably abrogated H3K27me3 in promoter regions of target genes tested (Supplementary Figure S4C and D).

In addition, when we analyzed occupancy of SET/TAF-Iβ, RING1B and H2AK119ub in promoter regions using published chromatin immunoprecipitation sequencing (ChIP-seq) data (GSE54580) (25) and our unpublished data, we were able to find out that SET/TAF-Iβ and RING1B occupied different regions of target gene promoter and transcription start site (TSS) area overall (Supplementary Figure S4E and F). Specifically, *CDKN1A* shows dramatic regional differences in SET/TAF-Iβ and RING1B occupancies (more upstream of the TSS occupancy by SET/TAF-Iβ and more downstream occupancy by RING1B) which suggests that H2AK119ub in *CDKN1A* promoter region is regulated by SET/TAF-Iβ-MIB1 complex rather than PRC1 (Supplementary Figure S4E). Altogether, these findings suggest that SET/TAF-Iβ and MIB1 regulate H2AK119ub in their target gene promoters to regulate transcription.

### Knockdown of MIB1 or SET/TAF-Iβ impairs proliferation of HCT116 cells by inhibiting DNA replication and cell cycle progression

**Figure 7.**
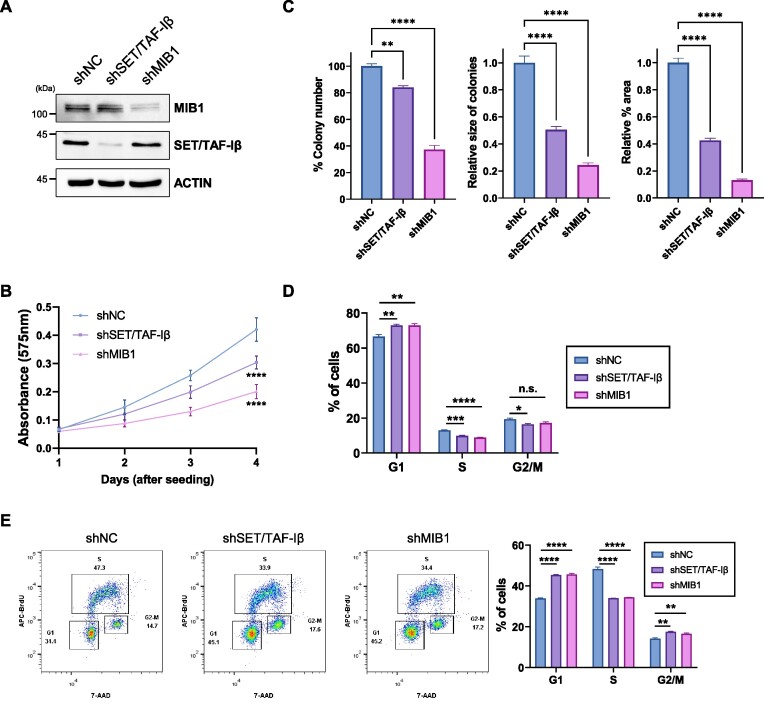
Knockdown of MIB1 impairs proliferation of HCT116 cells by inhibiting DNA replication. (**A**) Western blot analysis confirming the knockdown of MIB1 or SET/TAF-Iβ in HCT116 cells. (**B**) MTT assay to assess cell viability of HCT116 cells under indicated conditions. The *P*-values were determined using one-way ANOVA followed by Dunnett's multiple comparisons test. Data are expressed as mean ± SD (*n* = 6). ****P < 0.0001. (**C**) Quantification of colony formation assay results with MIB1 or SET/TAF-Iβ knockdown cells. The *P*-values were determined using one-way ANOVA followed by Dunnett's multiple comparisons test. Data are expressed as mean ± SEM (*n* = 3). ****P < 0.0001 and **P < 0.01. (**D**) Cell cycle analysis with propidium iodide followed by FACS analysis. The *P*-values were determined using one-way ANOVA followed by Dunnett's multiple comparisons test. Data are expressed as mean ± SEM (*n* = 3). ****P < 0.0001, ***P < 0.001, **P < 0.01, *P < 0.05 and n.s., not significant. (**E**) BrdU incorporation assay followed by FACS analysis (*n* = 3). The *P*-values were determined using one-way ANOVA followed by Dunnett's multiple comparisons test. Data are expressed as mean ± SEM (*n* = 3). ****P < 0.0001 and **P < 0.01.

Since we found that SET/TAF-Iβ and MIB1 regulate DNA replication-related genes in HCT116 cells, we next examined the functional roles of these proteins in HCT116 cells. First, we tested proliferation of HCT116 cells deficient in SET/TAF-Iβ or MIB1 (Figure 7A). Knockdown of SET/TAF-Iβ or MIB1 substantially inhibited the proliferation of HCT116 cells as measured by the MTT assay and ectopic expression of these proteins partially recovered proliferation of HCT116 cells (Figure 7B and Supplementary Figure S5A). Consistently, when we observed proliferation of HCT116 cells for a longer time period by colony formation assay under the same conditions, SET/TAF-Iβ or MIB1 deficiency induced an inhibitory effect on the proliferation of HCT116 cells according to number, size and area of the colonies (Figure 7C and Supplementary Figure S5B).

Since we found, by transcriptome analysis, that knockdown of MIB1 impairs the expression of genes involved in DNA replication (Figure 5D), we performed cell cycle analysis with flow cytometry. Using propidium iodide (PI) to stain DNA in HCT116 cells, we observed that the ratio of cells in G1 phase increased, and the ratios of those in S phase and G2/M phase notably decreased upon SET/TAF-Iβ or MIB1 depletion (Figure 7D and Supplementary Figure S5C). To examine the distribution of cell cycle more precisely, we adopted 5′-bromo-2′-deoxyuridine (BrdU) incorporation assay followed by 7-amino-actinomycin D (7-AAD) staining. Importantly, percentage of cells in S phase of cell cycle was remarkably lower and cells were arrested in the G1 phase in SET/TAF-Iβ- or MIB1-knockdown cells (Figure 7E and Supplementary Figure S5D). These results indicate that MIB1 and SET/TAF-Iβ regulate DNA replication process and cell cycle progression in HCT116 cells.

Analysis of data from the GENT2 database revealed that MIB1 expression is elevated in colon cancer as compared with healthy colon tissues (Supplementary Figure S6A). Notably, expression levels of MIB1 were found to be positively correlated with colon cancer stages when analyzed with cBioPortal (45,46) (Supplementary Figure S6B). Moreover, survival analysis with GENT2 database revealed that patients with higher expression levels of MIB1 showed poor prognosis compared with patients with lower expression levels of MIB1 (Supplementary Figure S6C). These results, along with MIB1 knockdown RNA-seq profiling, strongly indicate that SET/TAF-Iβ and MIB1 have roles in regulating DNA replication and cell cycle progression and a possible synergistic role in governing these processes.

Taken together, our study demonstrates that SET/TAF-Iβ and MIB1 regulate H2AK119ub levels and affect transcription of genes related to DNA replication and cell cycle progression in HCT116 cells. Importantly, knockdown of MIB1 or SET/TAF-Iβ remarkably inhibited proliferation of HCT116 cells by impeding DNA replication and cell cycle progression, suggesting that MIB1 and SET/TAF-Iβ has a crucial role in proliferation of HCT116 cells.

## DISCUSSION

Mono-ubiquitination of histone H2A at lysine 119 is well-known for its role in transcriptional repression. In this report, we found that overexpression of SET/TAF-Iβ induces H2AK119ub in colon cancer cells. To analyze interaction partners of SET/TAF-Iβ in HCT116 cells, we performed IP assay followed by LC-MS/MS and found that MIB1, an E3 ligase, was one of the proteins interacting with SET/TAF-Iβ. Specifically, *in vitro* ubiquitination assay demonstrated that purified GST-SET/TAF-Iβ and GST-MIB1 were able to catalyse H2AK119ub. Moreover, RNA-seq analyses revealed that MIB1 and SET/TAF-Iβ regulates expression of genes related to DNA replication in HCT116 cells. In fact, knockdown of MIB1 strongly inhibited cell cycle progression and proliferation of HCT116 cells by suppressing the expression of genes required for DNA replication. Taken together, our data suggest that MIB1 acts as a novel binding partner of SET/TAF-Iβ to form ubiquitination complex and the complex has roles in regulating gene expressions at the transcriptional level through histone modification in colon cancer.

In humans, there are about 40 known E2 ubiquitin-conjugating enzymes (47). In order to assign SET/TAF-Iβ into a group of E2 enzymes, we analyzed the sequences of SET/TAF-Iβ and E2 enzymes. However, we could not find similarities between the amino acid sequences of SET/TAF-Iβ and other E2 enzymes. Besides, when we checked amino acid sequence of the SET/TAF-Iβ, there was no cysteine residue. However, there have been many studies about non-canonical ubiquitination besides lysine ubiquitination which suggest that conjugation of ubiquitin can be mediated by other residues including lysine, serine, threonine, or tyrosine. Therefore, we speculate that SET/TAF-Iβ might conjugate ubiquitin via non-canonical mechanism (34,48–51). Also, we showed that the ubiquitin can be attached to SET/TAF-Iβ by performing *in vitro* ubiquitination assay without substrate (Figure 4E). The shifted band which represents ubiquitin-attached GST-SET/TAF-Iβ was only detected when E1 was added. The fact that INHAT domain of SET/TAF-Iβ was dispensable for E2 enzyme activity and our *in vitro* ubiquitination assay with deletion mutants suggest the possibility of ubiquitin-binding domain locates between amino acids 100 and 225 of SET/TAF-Iβ.

Recent study suggests that E3 ligase NEDD4 ubiquitinates histone H3 and recruits GCN5 for subsequent H3 acetylation upon glucose induction (52). Another study indicates that CBX8 and SET/TAF-Iβ co-bind to the tumor suppressor *SUSD2* promoter to establish H2AK119ub and prevent the acetylation of histone H3, resulting in transcriptional suppression of *SUSD2* (53). Since SET/TAF-Iβ has been known for its INHAT activity, it is reasonable to speculate that any genomic loci where SET/TAF-Iβ is recruited might represent hypoacetylation of histones. Our study provides evidence that SET/TAF-Iβ INHAT domain is not required for its H2AK119ub-related activity suggests it targets different transcriptome for reprograming.

The importance of H2AK119ub through the E3 ligase activity of RING1A/B in PRC1-mediated gene repression has been studied intensively including target genes (54–56). Loss of RING1B display major depletion of mono-ubiquitinated H2A and eventually leads to a full loss of PRC1 silencing resulting in early embryonic lethality (57). We speculate that SET/TAF-Iβ-MIB1-mediated H2AK119ub has its own specific target genes, which suggested in our study, governing DNA replication, cell cycle regulation, and proliferation.

In fact, when we compared downregulated genes in shMIB1 HCT116 and siSET/TAF-Iβ U2OS cells, 27 overlapping genes were found (Figure 6C). Among these 27 genes, genes which are known to be important in DNA replication process, DNA synthesis, and cell cycle were included, such as *RAD51*, *CDK14*, *GINS1*, *NCAPG*, *RFC5*, *DTL*, *CCNA2*, *YES1*. Also, there were 66 upregulated genes which might be regulated by SET/TAF-Iβ-MIB1-mediated H2AK119ub (Figure 6B).

*MIB1* is a gene that encodes a protein that functions as an E3 ubiquitin ligase. The protein positively regulates Notch signaling pathway via ubiquitination of the Notch receptors (58). Previous result suggests that homozygous *Mib1* deletion in mice resulted in lethality by embryonic day 10.5 due to severely reduced Notch signaling (59). Conditional knockout study found that *Mib1* had a role in neurogenesis and gliogenesis in developing mouse spinal cord (60). Various *MIB1* mutations including missense, nonsense, silent mutations and frameshift deletions and insertions are observed in different cancers. The study suggests that MIB1 mutation is present in 1.39% of AACR GENIE cases, with colon, lung adenocarcinoma and astrocytoma and colorectal adenocarcinoma having the greatest prevalence of mutation (Supplementary Figure S6D) (61).

Inhibiting DNA synthesis is considered to be one of the important therapeutic strategies to treat various diseases including cancer and has been studied widely (62). Our findings indicate that SET/TAF-Iβ and MIB1 regulate the expression of genes related to DNA replication by mediating H2AK119ub. Indeed, our study shows that knockdown of SET/TAF-Iβ or MIB1 delayed cell cycle progression and proliferation of HCT116 cells. All together, these findings suggest that SET/TAF-Iβ and MIB1 might provide novel mechanism for identifying therapeutic targets for colon cancer.

It is intriguing that overlapping target genes related with DNA replication and cell cycle progression identified by transcriptome analyses of MIB1 and SET/TAF-Iβ depletion showed their promoter H2AK119ub levels down-regulated in both transcriptionally upregulated and downregulated target genes. The fact that overlapping target genes with their promoter H2AK119ub levels downregulated and still transcriptionally repressed suggests another mechanism by which SET/TAF-Iβ-MIB1 regulate their target genes. In fact, genome-wide ChIP-seq analysis in mouse embryonic stem cells showed unexpected result that only 31% of canonical PRC1 (cPRC1) and non-canonical PRC1 (ncPRC1) target genes overlap which suggests the presence of transcriptionally active target genes by PcG (63). It is also striking that the different levels of H2AK119ub between nPRC1 and ncPRC1 target genes were found which strongly suggest a complex pattern of gene expression is exist according to the specific set of biological functions (63). In fact, another study revealed that ncPRC1.1 targets active genes independent of PRC2-mediated H3K27me3 in leukemogenesis (25).

The fact that knockdown of MIB1 enhanced H4 acetylation level in ChIP-qPCR assay suggests an interesting role of MIB1 in regulating histone modifications and transcription. Knockdown of SET/TAF-Iβ and resulting loss of H3K27me3 in promoter of target genes can be expected since the crosstalk between H2AK119ub and H3K27 methylation and further transcriptional repression has been well characterized. In addition, SET/TAF-Iβ has been known for its transcriptional repressive characteristic. However, more experiments are needed to delineate the detailed regulatory mechanism between SET/TAF-Iβ-MIB1-mediated H2AK119ub and H3K27me3.

ChIP-seq analyses of SET/TAF-Iβ, RING1B and H2AK119ub in one of the overlapping target gene, *CDKN1A* promoter region using published (GSE54580) (25) and our unpublished data show the clear different occupancies between RING1B and SET/TAF-Iβ which strongly supports that SET/TAF-Iβ and MIB1-mediated H2AK119ub is PRC1-independent.

In summary, we report another layer of epigenetic regulatory mechanism by multitasking acidic protein SET/TAF-Iβ. We show that one of the important epigenetic markers, H2AK119ub is regulated by SET/TAF-Iβ-MIB1 ubiquitinating complex in various cancers including colon cancer in PRC1-independent mechanism. Altogether, our results suggest the existence of specific transcriptome programming regulated by SET/TAF-Iβ-MIB1-mediated H2AK119ub.

## Supplementary Material

zcad050_Supplemental_File

## Data Availability

All MS data and search result files have been deposited by the Proteomics Data Curation Center at KBSI into the ProteomeXchange Consortium through the MassIVE Partner Repository with the identifier PXD038806 for ProteomeXchange and MassIVE MSV000090898 for MassIVE. The RNA-seq datasets generated and analyzed in this study are available and can be downloaded from NCBI Gene Expression Omnibus (GEO) under accession number GSE218399. The ChIP-seq and RNA-seq datasets analyzed in this study were obtained from NCBI GEO under accession number GSE54580 and GSE83635, respectively.
