# Supplementary material for: INHAT subunit SET/TAF-Iβ regulates PRC1-independent H2AK119 mono-ubiquitination via E3 ligase MIB1 in colon cancer
Source: NAR Cancer. 2023 Sep 22;5(3):zcad050. doi: 10.1093/narcan/zcad050 (PMC10516711; doi:10.1093/narcan/zcad050)
Supplement: zcad050_Supplemental_File [file zcad050_supplemental_file.pdf]

Supplementary Data for

INHAT subunit SET/TAF-Iβ regulates PRC1 independent H2AK119 mono-ubiquitination via E3 ligase MIB1 in colon cancer

Junyoung Park[1,†], Ji-Young Kim[1,†], Jin Woo Park[1], Joo Young Kang[1], Hyein Oh[1], Ja Young Hahm[1], Yun-Cheol Chae[1], Debabrata Chakravarti[2] and Sang Beom Seo[1,*]

[1]Department of Life Science, College of Natural Sciences, Chung-Ang University, Seoul 06974, Republic of Korea

[2]Division of Reproductive Sciences in Medicine, Department of Obstetrics and Genecology, Northwestern University Feinberg School of Medicine, Chicago, IL 60611, USA

*Corresponding author: Sang Beom Seo (sangbs@cau.ac.kr)

Supplementary Figure S1. Overexpression of SET/TAF-Iβ induces H2AK119ub in multiple cell lines

Supplementary Figure S2. MIB1 regulates H2AK119ub with SET/TAF-Iβ but TRIM21 doesn't affect H2AK119ub in HCT116 cells

Supplementary Figure S3. SET/TAF-Iβ does not interact with and PRC1 subunits and SET/TAF-Iβ and MIB1 does not affect protein level of RING1B in HCT116 cells

Supplementary Figure S4. Knockdown of SET/TAF-Iβ or MIB1 affects acetylation of histone H4 and tri-methylation of histone H3 at their target gene promoters in HCT116 cells

Supplementary Figure S5. Knockdown of SET/TAF-Iβ or MIB1 delays cell cycle progression

Supplementary Figure S6. MIB1 is upregulated in colon cancer and associated with poor prognosis

Supplementary Figure S1

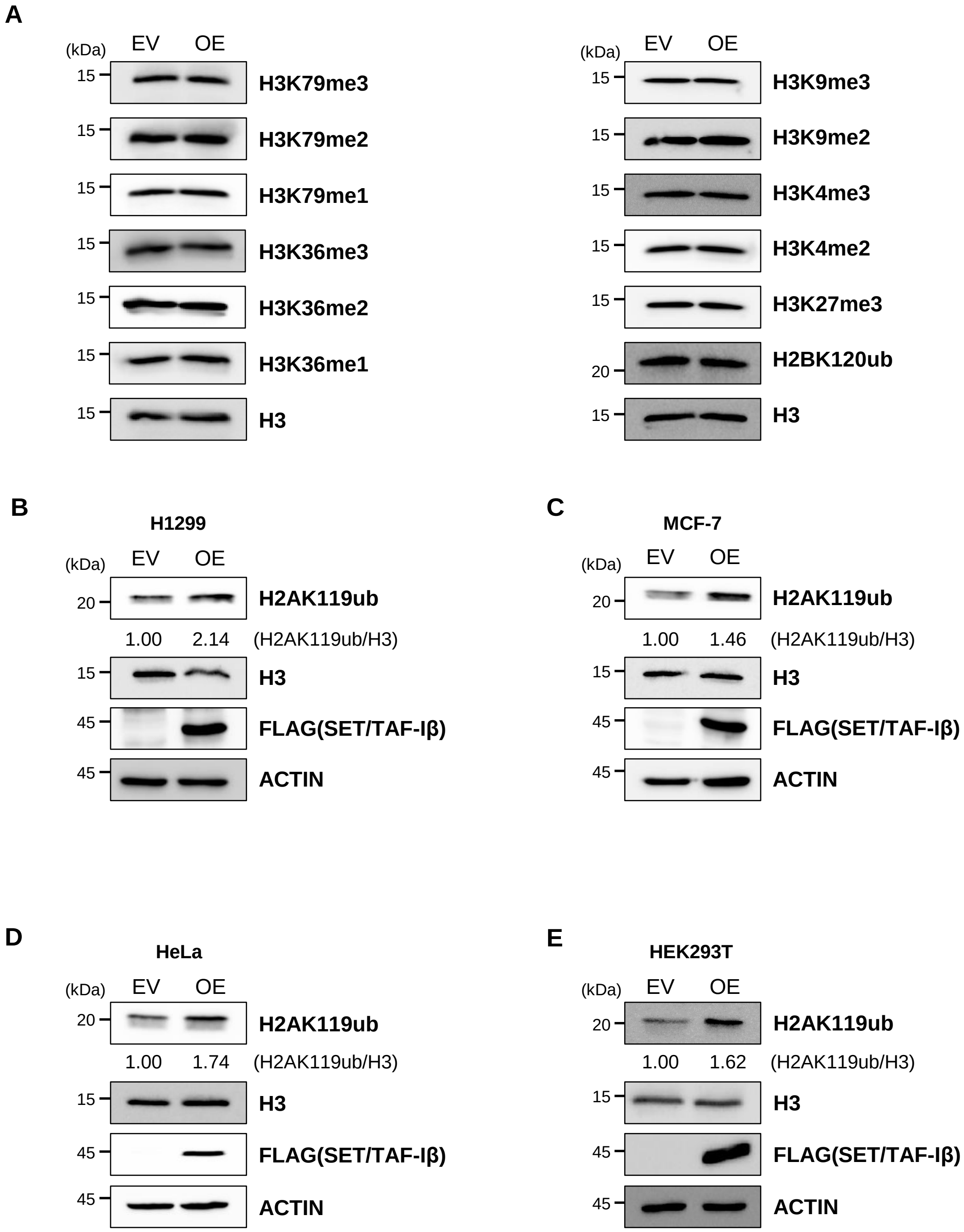

Supplementary Figure S1. Overexpression of SET/TAF-Iβ induces H2AK119ub in multiple cell lines

(A) Levels of various histone modifications were assessed by western blotting after overexpressing SET/TAF-Iβ in HCT116 cells. EV, empty vector; OE, overexpression.

(B) H2AK119ub levels in H1299 cells overexpressing SET/TAF-Iβ were examined by western blotting.

(C) H2AK119ub levels in MCF-7 cells overexpressing SET/TAF-Iβ were examined by western blotting.

(D) H2AK119ub levels in HeLa cells overexpressing SET/TAF-Iβ were examined by western blotting.

(E) H2AK119ub levels in HEK293T cells overexpressing SET/TAF-Iβ were examined by western blotting.

Supplementary Figure S2

A

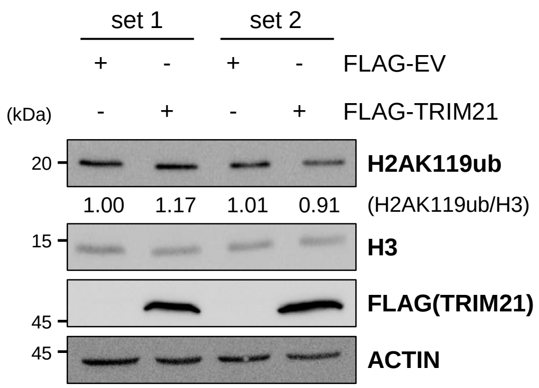

B

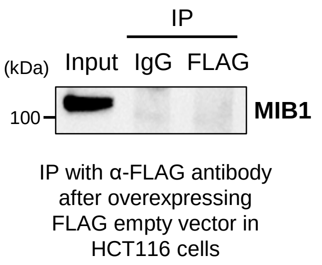

C

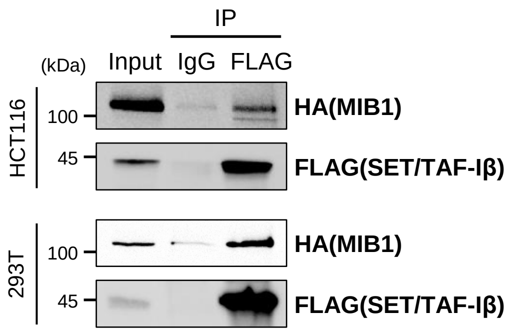

D

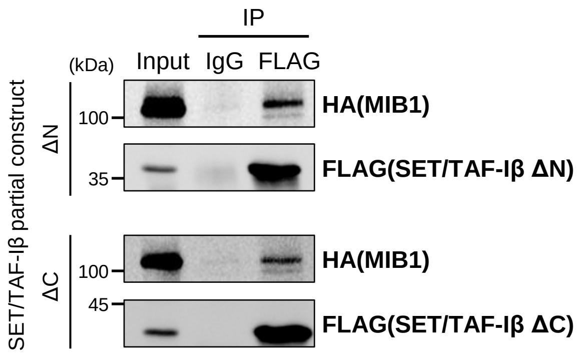

E

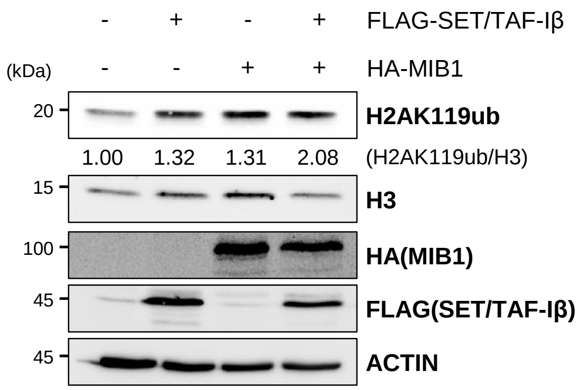

F

Histone	Gene	Coverage (%)	Counts
H4	HIST1H4A	40.80%	5
H2A.Z	H2AFZ	25.80%	3
H2A.V	H2AFV	25.80%	3
H2A type 1	HIST1H2AG	21.50%	2
H2A.J	H2AFJ	21.70%	2
H2A type 1-J	HIST1H2AJ	21.90%	2
H2A type 1-H	HIST1H2AH	21.90%	2
H2A type 2-A	HIST2H2AA3	21.50%	2
H2A type 2-C	HIST2H2AC	21.70%	2
H2A type 1-D	HIST1H2AD	21.50%	2
H2B type 1-J	HIST1H2BJ	19.80%	4
H2B type 3-B	HIST3H2BB	19.80%	4
H2B type 2-E	HIST2H2BE	19.80%	4
H2B type 1-B	HIST1H2BB	19.80%	4
H2B type 1-O	HIST1H2BO	19.80%	4
H2B type 1-K	HIST1H2BK	19.80%	5
H2B type 1-L	HIST1H2BL	19.80%	5
H2B type 1-M	HIST1H2BM	19.80%	5
H2B type 1-N	HIST1H2BN	19.80%	5
H2B type 1-H	HIST1H2BH	19.80%	5
H2B type 2-F	HIST2H2BF	19.80%	5
Isoform of H2B type 2-F	HIST2H2BF	18.70%	5
H2B type 1-C/E/F/G/I	HIST1H2BC	19.80%	5
H2B type 1-D	HIST1H2BD	19.80%	5
H2B type F-S	H2BFS	19.80%	5
H1.4	HIST1H1E	10.50%	2
H1.2	HIST1H1C	10.80%	2
H1.3	HIST1H1D	10.40%	2
H1.5	HIST1H1B	10.20%	2

Supplementary Figure S2. MIB1 regulates H2AK119ub with SET/TAF-Iβ but TRIM21 doesn't affect H2AK119ub in HCT116 cells

(A) H2AK119ub levels in cells overexpressing TRIM21 were examined by western blotting.

(B) Western blot analysis after immunoprecipitation (IP) with FLAG antibody after overexpressing FLAG empty vector in HCT116 cells.

(C) Interaction between SET/TAF-Iβ and MIB1 was examined by complex-immunoprecipitation (co-IP). HCT116 and 293T cells overexpressing both FLAG-SET/TAF-Iβ and HA-MIB1 were used.

(D) Interactions between SET/TAF-Iβ partial constructs and MIB1 were examined by co-IP. HCT116 cells overexpressing both FLAG-SET/TAF-Iβ partial construct and HA-MIB1 were used.

(E) H2AK119ub levels in cells overexpressing SET/TAF-Iβ or MIB1 were examined by western blotting.

(F) List of histone proteins obtained from immunoprecipitation followed by liquid chromatography with tandem mass spectrometry (LC-MS/MS) analysis. Sequence coverage and count for each histone are indicated.

Supplementary Figure S3

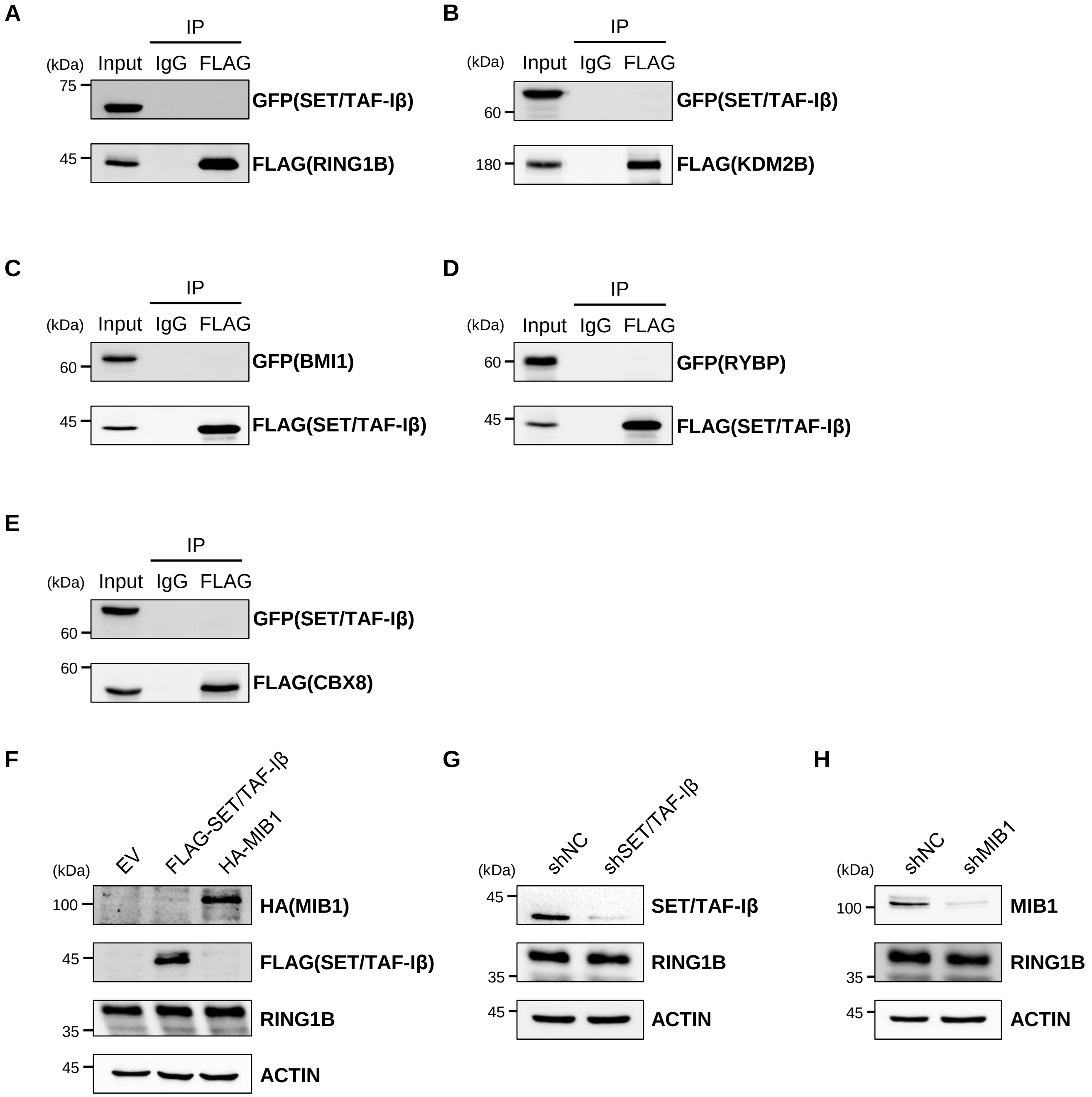

Supplementary Figure S3. SET/TAF-Iβ does not interact with and PRC1 subunits and SET/TAF-Iβ and MIB1 does not affect protein level of RING1B in HCT116 cells

(A) Interaction between SET/TAF-Iβ and RING1B in HCT116 cells was assessed by co-IP with ectopic expression of both proteins.

(B) Interaction between SET/TAF-Iβ and KDM2B in HCT116 cells was assessed by co-IP with ectopic expression of both proteins.

(C) Interaction between SET/TAF-Iβ and BMI1 in HCT116 cells was assessed by co-IP with ectopic expression of both proteins.

(D) Interaction between SET/TAF-Iβ and RYBP in HCT116 cells was assessed by co-IP with ectopic expression of both proteins.

(E) Interaction between SET/TAF-Iβ and CBX8 in HCT116 cells was assessed by co-IP with ectopic expression of both proteins.

(F) Protein level of RING1B was assessed by western blotting after overexpression of SET/TAF-Iβ or MIB1 in HCT116 cells.

(G) Protein level of RING1B was assessed by wester blotting after depleting SET/TAF-Iβ in HCT116 cells.

(H) Protein level of RING1B was assessed by wester blotting after depleting MIB1 in HCT116 cells.

Supplementary Figure S4

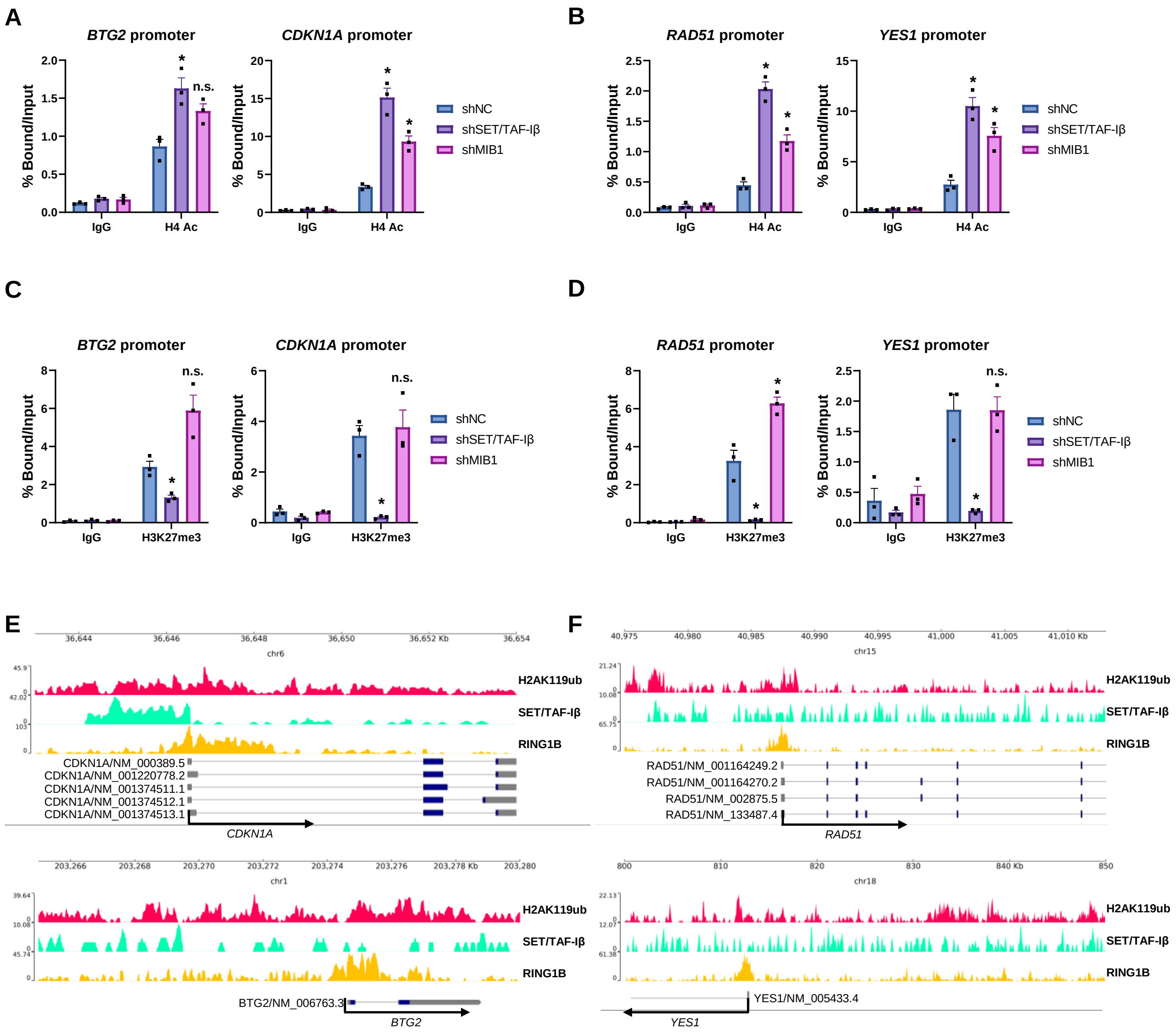

Supplementary Figure S4. Knockdown of SET/TAF-Iβ or MIB1 affects acetylation of histone H4 and tri-methylation of histone H3 at their target gene promoters in HCT116 cells

(A) Chromatin immunoprecipitation quantitative real-time PCR (ChIP-qPCR) assay to evaluate acetylated H4 (H4 Ac) level in promoters of upregulated DEGs. The *P*-values were determined using one-way ANOVA followed by Dunnett's multiple comparisons test. Data are expressed as mean ± SEM (n = 3). $^*P < 0.05$ and n.s., not significant.

(B) ChIP-qPCR assay to evaluate H4 Ac level in promoters of downregulated DEGs. The *P*-values were determined using one-way ANOVA followed by Dunnett's multiple comparisons test. Data are expressed as mean ± SEM (n = 3). $^*P < 0.05$.

(C) ChIP-qPCR assay to evaluate tri-methylated H3K27 (H3K27me3) level in promoters of upregulated DEGs. The *P*-values were determined using one-way ANOVA followed by Dunnett's multiple comparisons test. Data are expressed as mean ± SEM (n = 3). $^*P < 0.05$ and n.s., not significant.

(D) ChIP-qPCR assay to evaluate H3K27me3 level in promoters of upregulated DEGs. The *P*-values were determined using one-way ANOVA followed by Dunnett's multiple comparisons test. Data are expressed as mean ± SEM (n = 3). $^*P < 0.05$ and n.s., not significant.

(E) Custom track plot of chromatin immunoprecipitation sequencing (ChIP-seq) data with indicated antibodies. Anti-H2AK119ub and anti-RING1B ChIP-seq data were obtained from GSE54580 and anti-SET/TAF-Iβ ChIP-seq data were obtained from our unpublished data. Promoter and transcription start site (TSS) regions of *CDKN1A*, *BTG2* were analyzed.

(F) Custom track plot of ChIP-seq data with indicated antibodies. Promoter and TSS regions of *RAD51*, *YES1* were analyzed.

Supplementary Figure S5

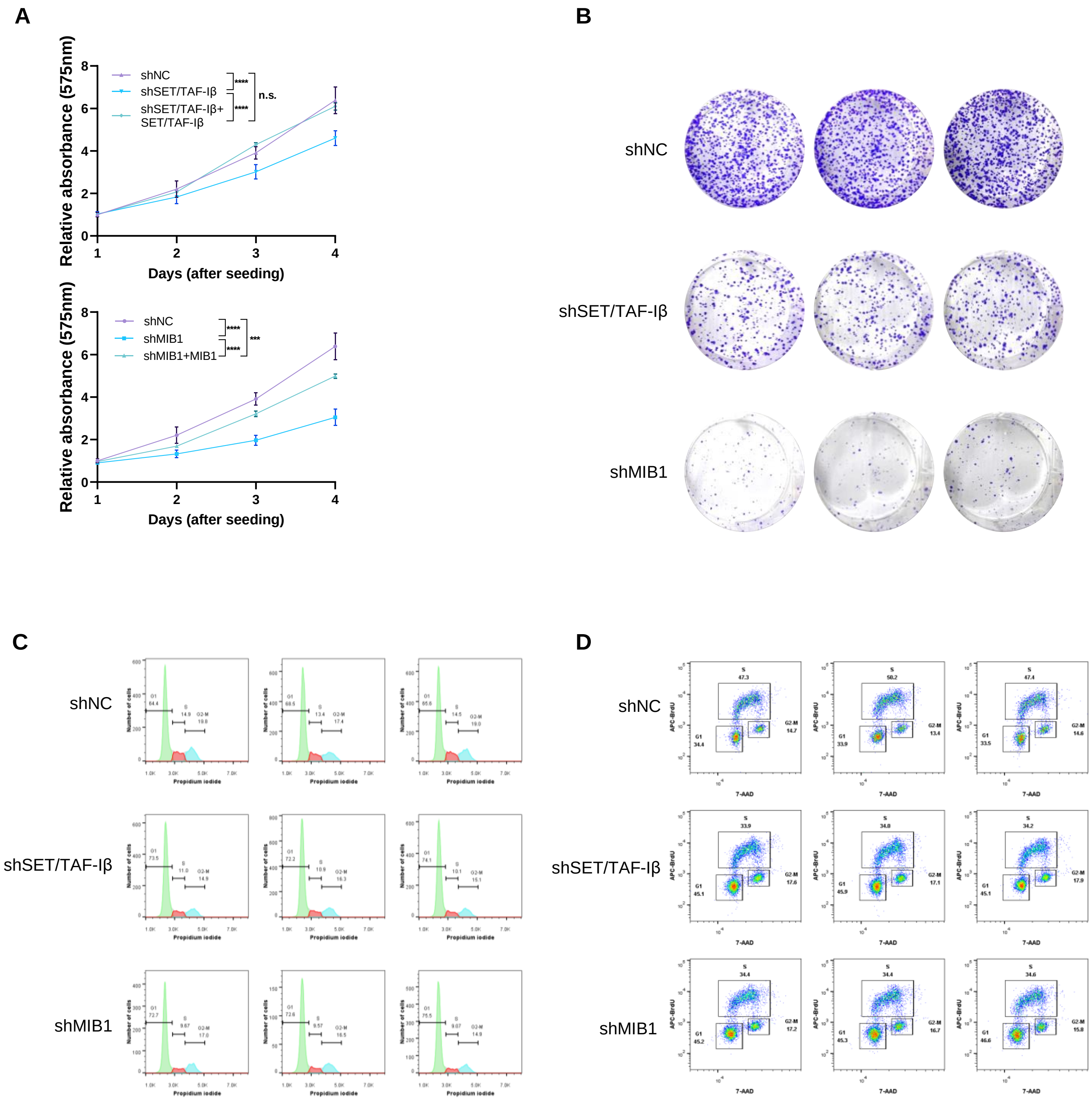

Supplementary Figure S5. Knockdown of SET/TAF-Iβ or MIB1 delays cell cycle progression

(A) Cell viability assessment with MTT assay with knockdown cells and recovered cells. The *P*-values were determined using one-way ANOVA followed by Dunnett's multiple comparisons test. Data are expressed as mean ± SD (n = 6). ****$P < 0.0001$, ***$P < 0.001$ and n.s., not significant.

(B) Colony formation assay result with control and knockdown cells. Quantified data is presented as a bar graph in Figure 7C.

(C) Fluorescence-activated cell sorting (FACS) analysis showing distribution of cells in each cell cycle. Propidium iodide was used for staining of DNA. These results are presented as a bar graph in Figure 7D.

(D) BrdU incorporation assay followed by FACS analysis. These results are presented as a bar graph in Figure 7E.

Supplementary Figure S6

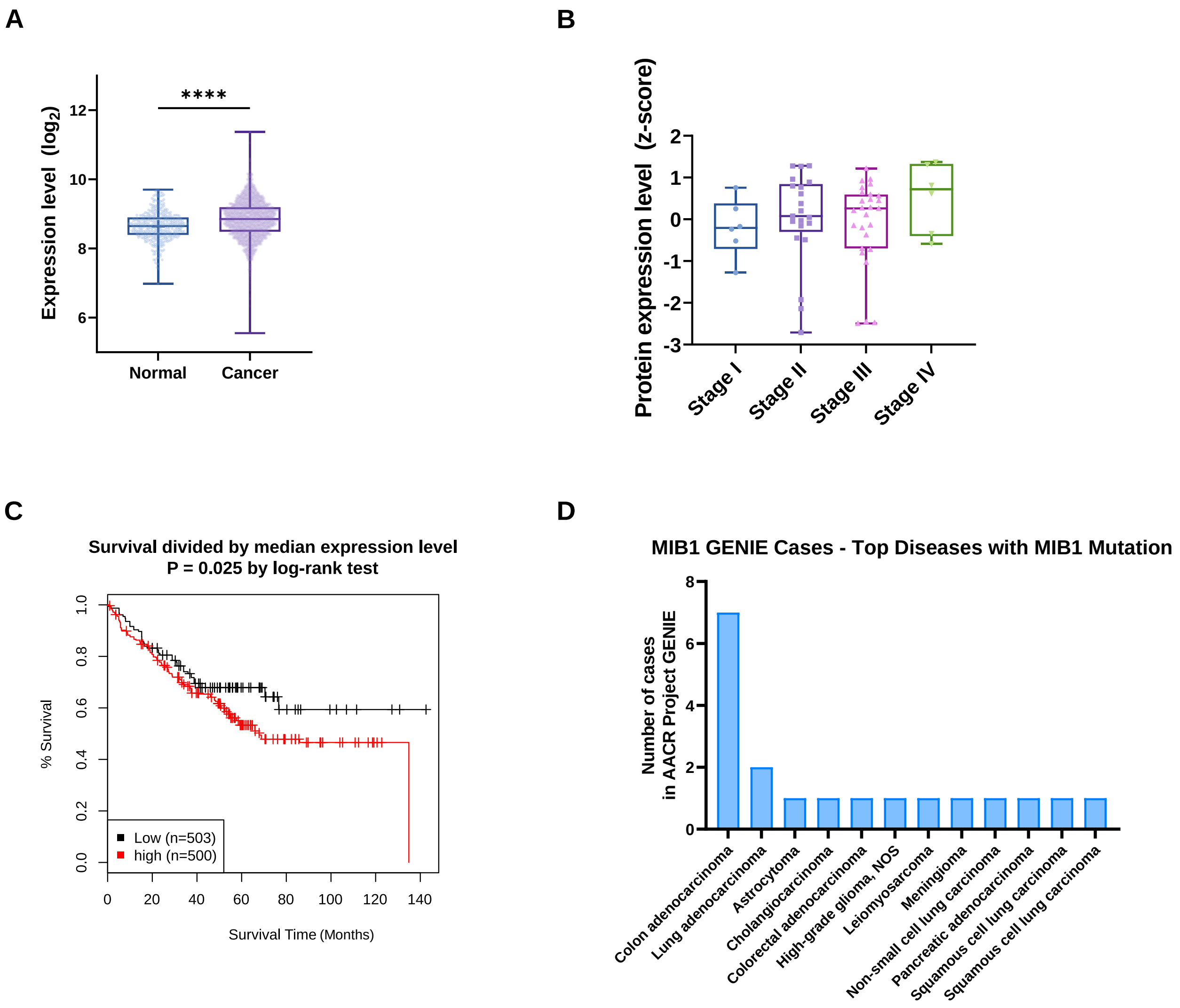

Supplementary Figure S6. MIB1 is upregulated in colon cancer and associated with poor prognosis

(A) Expression levels of MIB1 in normal (n = 397) and cancer (n = 3775) colon tissues were analyzed using the data available in the GENT2 database. The *P*-value was calculated with unpaired t test with Welch's correction. The boxes extend from the 25th to the 75th percentiles and the line in the middle of the box indicates the median value. ****P < 0.0001.

(B) Protein expression levels of MIB1 in colon cancer were examined in each stage of cancer (n = 6, 21, 27 and 6, in stages I–IV, respectively) using cBioportal. The boxes extend from the 25th to the 75th percentiles and the line in the middle of the box indicates the median value.

(C) Survival rates according to expression levels of MIB1 in colon cancer patients were analyzed using the data available in the GENT2 database. The *P*-value was calculated with log-rank test.

(D) Bar graph indicating top diseases with MIB1 mutation from AACR Project GENIE.
